# Rapid warming in South America during the last deglaciation

**DOI:** 10.1038/s41467-026-74093-x

**Published:** 2026-06-20

**Authors:** A. Ampuero, N. M. Stríkis, A. N. Meckler, Y. Krüger, M. Vuille, H. Vonhof, L. Pasqualetto, V. C. Mayta, N. M. M. Medina, G. Utida, H. Zhang, H. Cheng, R. L. Edwards, L. E. P. Travassos, P. Piacsek, T. K. Akabane, F. W. Cruz

**Affiliations:** 1 Institute of Geosciences, University of São Paulo, São Paulo, Brazil; 2 Department of Climate Geochemistry, Max-Planck Institute for Chemistry, Mainz, Germany; 3 Department of Earth Science, University of Bergen, Bergen, Norway; 4 Bjerknes Centre for Climate Research, Bergen, Norway; 5 Department of Atmospheric and Environmental Sciences, University at Albany, State University of New York, Albany, NY USA; 6 Department of Atmospheric and Oceanic Sciences, University of Wisconsin, Madison, Wisconsin USA; 7 Institute of Astronomy, Geophysics and Atmospheric Sciences, University of São Paulo, São Paulo, Brazil; 8 Institute of Global Environmental Change, Xi’an Jiaotong University, Xi’an, China; 9 Department of Earth and Environmental Sciences, University of Minnesota, Minneapolis, MN USA; 10 Department of Geography, Pontifical Catholic University of Minas Gerais, Minas Gerais, Brazil

**Keywords:** Palaeoclimate, Climate change

## Abstract

Understanding tropical land temperature response to rising atmospheric CO_2_ in the past is crucial for better constraining future climate projections. However, the evolution of regional land temperatures on paleoclimate timescales remains uncertain due to the paucity of precise records. Here we reconstructed temperatures across the last deglaciation using nucleation-assisted microthermometry in a stalagmite from central-eastern South America. We show that cave temperatures increased by 5.8 ± 0.3 °C (2 standard errors of the mean, SEM) from the Last Glacial Maximum to the early Holocene, broadly tracking global atmospheric CO_2_ and Antarctic temperatures. Our results reveal an abrupt regional warming across the Antarctic Cold Reversal-Younger Dryas (ACR-YD) transition, linked to the weakening of the Atlantic Meridional overturning circulation (AMOC). Notably, the most rapid warming at our cave was still slower than projections of future long-term warming, highlighting the unprecedented nature of the current greenhouse gas forcing.

## Abstract (web summary)

This study presents a tropical temperature record across the last deglaciation using stalagmites from central-eastern South America. Tropical temperature broadly tracks atmospheric CO₂ and ocean circulation.

## Introduction

The last glacial termination (Termination I) is the most recent interval of abrupt, large-scale climate reorganization prior to the industrial period. During this time, rising atmospheric CO_2_ acted as critical feedback, amplifying global warming that ultimately led to the collapse of the glacial climate system^1,2^. While CO_2_-driven warming is ubiquitous and defines the underlying structure of deglacial global temperature^1^, it is punctuated by millennial excursions concomitant with cold events in the Northern Hemisphere, such as Heinrich Stadial 1 (HS1) and Younger Dryas (YD); and in Antarctica, the Antarctic Cold Reversal (ACR). These events are linked with periods of weakening and strengthening in the Atlantic Meridional Overturning Circulation (AMOC), respectively.

The collapse of the AMOC disrupts the cross-equatorial heat transport, leading to an energy imbalance between the Northern and Southern Hemispheres^3^. To compensate for this thermal asymmetry, the atmospheric circulation reorganizes, resulting in the southward migration of the Intertropical Convergence Zone (ITCZ)^3^ and changes in the monsoon circulation^4,5^ with profound consequences for tropical ecosystems^6,7^. A key feature of this reorganization is the southward shift and intensification of the Southern Hemisphere westerly winds. The strengthening of the southern westerlies enhances upwelling and ventilation in the Antarctic zone of the Southern Ocean, prompting sustained CO_2_ outgassing, a prominent feedback linking AMOC and atmospheric CO_2_^2,8–13^. Indeed, global climate reanalysis supports the notion that radiative forcing combining greenhouse gases (primarily CO_2_) and ice-sheet albedo feedbacks explains more than 90% of the mean global surface temperature evolution during the deglaciation, while 3.5% can be attributed to AMOC-driven bipolar seesaw dynamics and orbitally-controlled high-latitude insolation changes^14^.

While understanding the Earth’s climate system’s sensitivity to rising CO_2_ levels is a central tenet of climate change science, the response of tropical temperatures remains poorly constrained during the last deglaciation due to the paucity of paleotemperature records and the complexity and model dependence of the feedback mechanisms involved. In this context, the deglacial period provides a valuable framework for examining the climate’s response to CO_2_ forcing on both global and regional scales. A robust understanding of the land-surface response to rising CO₂ requires temperature records with well-resolved chronologies. Yet, the scarcity of geological archives suitable for paleothermometry poses a major challenge in tropical regions, where precise temperature reconstructions are rare and often bear uncertainties exceeding 2 °C.

**Fig. 1:**
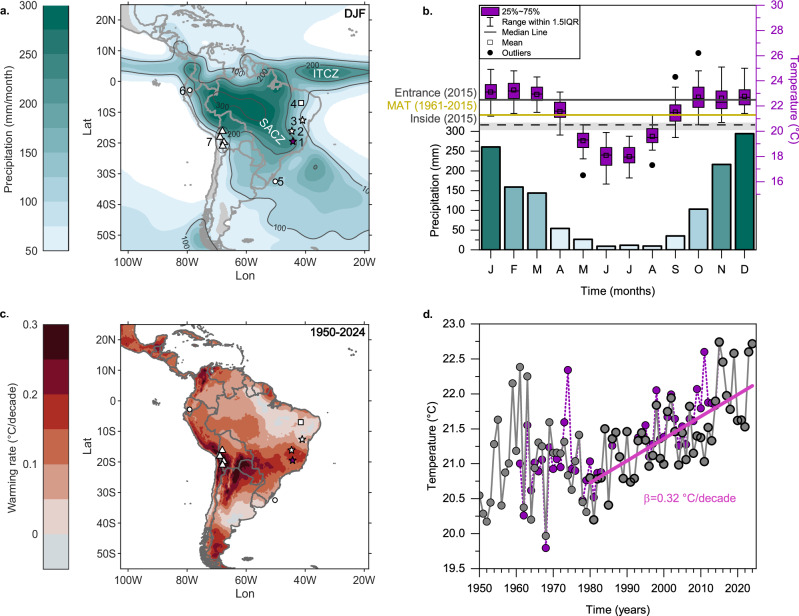
Geographic setting and study site location. **a** Map of austral summer precipitation with data from the Global Precipitation Measurement Mission (GPM, 2001–2020)^64^ during the peak monsoon season (December–January–February), with the locations of various paleoclimate records from speleothems (stars), sediment cores (circles), groundwater (square) and glaciers (triangles) referenced in this study: 1 – Rei do Mato (this study), 2 – Lapa Sem Fim^5^, 3 – Paixao^5^, 4 – noble gas record^15^, 5 – core GeoB6211-2^16^, 6 – lake Llaviucu^20^ and, 7 – glacier equilibrium lines^22^; **b** Precipitation (bars) and surface air temperature at 2 m (boxplot) observed near the cave site at Sete Lagoas station (1961–2015) (INMET: OMM 83586), average temperatures monitored in this study during 2015 at the cave entrance (solid gray line), inside the cave (dashed gray line) with a seasonal range from 20.1 to 20.7 °C (light gray shading area), and multi-decadal average surface air temperature from Sete Lagoas station (1961–2015, yellow line); **c** Warming rates for South America from 1950 to 2024 based on data from ERA 5 T2m reanalysis^65^ and **d** mean annual temperatures at the Rei do Mato from ERA 5 T2m reanalysis^65^ (gray line and circles). Warming trend from 1980 to 2024 (pink line). Reanalysis data is largely consistent with local temperatures observed at Sete Lagoas station (purple dot line and circles). ITCZ Intertropical Convergence Zone, SACZ South Atlantic Convergence Zone. Data from ERA5 is available by the CC-BY 4.0 license https://creativecommons.org/licenses/by/4.0/.

**Fig. 2:**
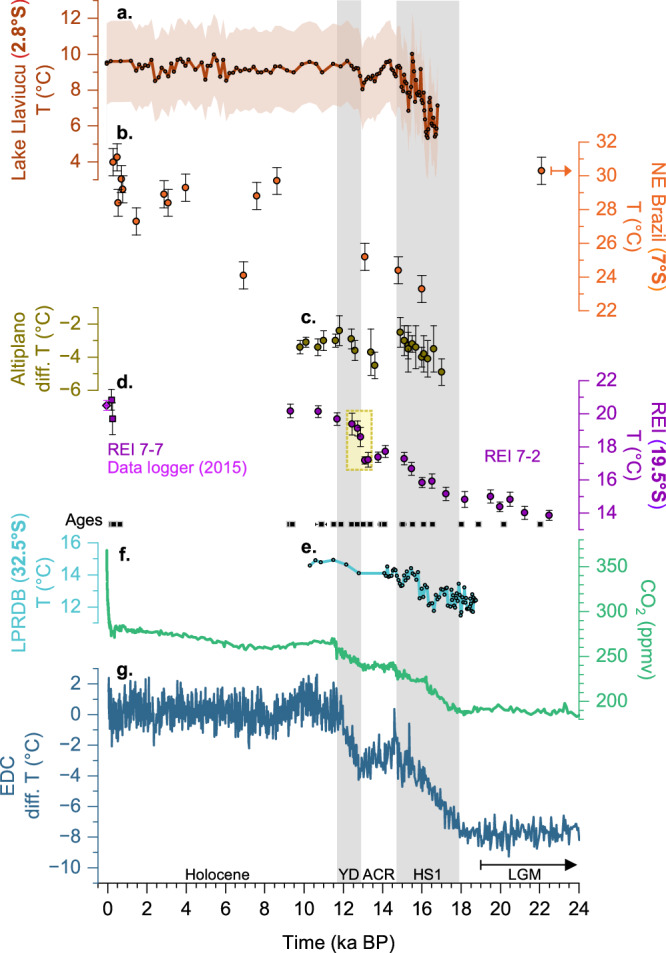
Deglacial temperature reconstructions in South America compared with Antarctic temperature and global atmospheric CO_2_. Proxy-based temperature reconstructions in South America from **a** Laguna Llaviucu derived from organic biomarkers^20^ with error bar of ±2.26 °C from the calibration; **b** Northeastern Brazil derived from noble gases in groundwater with ±0.8 °C error bars, where the orange arrow indicates that the date is likely older^15^; **c** Temperature relative to present from glacier equilibrium lines from the Andes, within the Altiplano region of Bolivia^22^ with propagated uncertainty error bars; **d** Rei do Mato microthermometry temperatures with 2SEM error bars from stalagmite REI 7-2 (purple circles) and measured at the top of stalagmite REI 7-7 (purple squares), average cave temperature measured in 2015 (20.5 °C, magenta diamond) with error bars representing the seasonal amplitude of ±0.3 °C and U-Th dates (black squares) (this study). The yellow dashed rectangle indicates the interval of most abrupt warming; **e** La Plata River Drainage Basin (LPRDB) GeoB6211-2 derived by methylation of branched tetraether (MBT’)- and cyclization of branched tetraether (CBT)-based mean air temperature^16^. Antarctic records of **f** CO_2_ concentration^66^ and **g** temperature anomalies at EPICA Dome C (EDC) site^21^. Gray vertical bars indicate Northern Hemisphere cooling episodes. YD Younger Dryas, HS1 Heinrich Stadial 1, ACR Antarctic cold reversal, LGM Last Glacial Maximum.

In the South American lowlands, the few available temperature reconstructions provide contrasting scenarios. While benchmark groundwater noble gas thermometry records from northeastern Brazil indicate 5.4 ± 0.6 °C warming between the Last Glacial Maximum (LGM) and the Holocene^15^, terrestrial biomarkers in a marine sediment core retrieved off southeastern Brazil suggest only ~2.5 °C warming^16^ (Figs. 1 and 2). In the tropical Andes, reconstructions of glacier equilibrium line altitude (ELA) in Peru and Bolivia indicate that LGM temperatures were ~6 °C lower than during the Holocene^17^. Pollen records from the western Amazon point out that during the last glacial period, temperatures were 4.5–6 °C cooler than today, allowing cold-adapted Andean species to thrive in the lowlands despite the continued presence of the rainforest^18,19^. Estimates based on biomarker data from lake sediments in the Andes of Ecuador suggest a ~4 °C change over this period^20^ (Figs. 1 and 2). Apart from discrepancies in the amplitude of deglacial warming and the contrasting geographic settings (lowlands and the Andes), the evolution of tropical South American temperature is in most cases broadly consistent with that observed in Antarctica^15,16,20–23^ (Fig. 2). An exception was found in the Colombian Andes, where temperature reconstructed from Lake Fúquene (5°N) pollen was suggested to align with the Greenland/North Atlantic millennial events^24^. While these records capture key features of the deglacial temperature evolution, the absolute magnitudes, spatial patterns, chronology, and variability at millennial timescale thus remain poorly constrained.

Here we employ an exceptionally precise paleothermometer on a well-dated archive to obtain a temperature record covering central-eastern South America, a tropical region particularly vulnerable to aridity, rising temperatures, and increasing intensity and frequency of heat extremes^25–27^. By applying nucleation-assisted microthermometry^28^ to stalagmite fluid inclusions from Rei do Mato Cave (Methods; Supplementary Text 2, Supplementary Figs. 3–6), we provide a high-precision cave temperature record that is closely related to land surface temperature, without relying on empirical calibrations^29,30^ as required for other temperature proxy records.

## Results and discussion

### The context of the study site and cave temperature

Rei do Mato Cave (19.50°S; 44.28°W; 800 m above sea level) is a tourist cave located in southeastern Brazil, Minas Gerais State, Sete Lagoas Municipality (Fig. 1). The site lies in the Brazilian Cerrado, a savanna-type vegetation characteristic of tropical seasonal climates. Mean annual rainfall is 1300 mm, with 90% of it falling between October and March, when the South American Monsoon System (SAMS) is active^31^ (Fig. 1a, b). The cave lies near the axis of the South Atlantic Convergence Zone (SACZ), a northwest-southeast-oriented convective band extending from the central Amazon to southeastern Brazil, constituting one of the main features of the SAMS^32–35^.

The cave is located on a hillside, with a single entrance on the upper level, and consists of a large hall that extends about 220 meters downward toward the innermost section, which is ~30 m below the entrance level (Supplementary Fig. 1). The overlaying bedrock does not exceed 30 m. In 2009, the stalagmite sample REI 7-2 was found broken in a pile of speleothems located near the end of the tourist walkway that traverses the entire hall (Supplementary Fig. 1). Although the exact location where the stalagmite formed is unknown, we assume it originally formed near where it was collected.

Present-day average temperature recorded from 1961 to 2015 in the nearby Sete Lagoas station (19.48°S;44.17°W; 753 m above sea level; station code: OMM 83586, Instituto Nacional de Meteorologia - INMET) is 21.3 °C, increasing at a rate of 0.32 °C per decade since 1980 (Fig. 1d). Cave monitoring from January 2015 until July 2016 revealed approximately stable conditions characterized by seasonal fluctuations of cave air temperatures within a range of 0.6 °C; and relative humidity near 100% during the rainy season and 94% during the dry season (Supplementary Text 1 and Supplementary Fig. 2). Continuous and simultaneous measurements inside and outside the cave were only obtained for 2015, revealing colder air temperatures inside the cave (20.5 °C) compared with the outside (22.5 °C) (Fig. 1b). While part of this 2 °C offset could be related to a delay of the transfer of the surface temperature signal into the cave^36,37^, the dominant factor is likely a cold-trap behavior related to the cave ventilation regime (Supplementary Text 1).

### Nucleation-assisted fluid inclusion microthermometry in speleothems

To reconstruct cave temperatures, we applied the nucleation-assisted fluid inclusion microthermometry method^38^, which allows the determination of the density of drip-water relics encapsulated within microscopic cavities in the stalagmite (i.e., fluid inclusions) by measuring the liquid-vapor homogenization temperature (T_h(obs)_). The measured T_h(obs)_ values are then corrected for the effect of surface tension^39^ yielding T_h∞_, the homogenization temperature of a hypothetical, infinitely large inclusion that, ideally, can be assumed to directly relate to the cave air temperature at the time the inclusion sealed off from the surrounding environment, thus providing a record of stalagmite formation temperatures (see Methods).

We determined stalagmite formation temperatures from 21 subsamples across different segments (layers) of stalagmite REI 7-2, selected according to the chronology profile. This provides a mean sampling resolution of ~600 years, covering the period from 22.5 to 9.3 ka BP (thousand years before present). Additionally, two temperatures measured at the top of a modern stalagmite (REI 7-7) collected near our deglacial record (REI7-2), match observed temperatures during 2015, supporting the accuracy of the microthermometry temperatures at Rei do Mato cave (Fig. 2d and Supplementary Fig. 5).

The thickness of the individual layers ranges from 1 to 5 mm, corresponding to intervals of 40–200 years based on the average stalagmite growth rate of ~26 µm/year (Supplementary Fig. 3). Each temperature data point in our cave record represents the mean of a near-Gaussian distribution of T_h∞_ values derived from ∼40 coeval fluid inclusions (Supplementary Fig. 4). The mean value of each T_h∞_ distribution is considered the best approximation of the stalagmite formation temperature, i.e., of the mean cave temperature of the time interval covered by the respective growth band. Standard deviations (1 SD) of the individual layers range from 0.7 to 2.1 °C (mean 1 SD: 1.3 °C). The precision of the reconstructed mean cave temperatures is reported as two standard errors of the mean (2SEM) and ranges from 0.2 °C to 0.7 °C (average 2SEM: 0.4 °C).

### Temperature evolution in the regional context

Our cave temperature reconstruction reveals a total deglacial warming of 5.8 ± 0.3 °C (2 SEM) from 14.4 ± 0.2 °C (2 SEM; *N* = 5) during full glacial conditions in the LGM to 20.1 ± 0.3 °C (2 SEM; *N* = 2) in the early Holocene (Fig. 2). This magnitude of warming is in close agreement with the noble gas-derived tropical record of northeastern Brazilian lowlands^15^ and is consistent with the general structure and timing of the temperature change observed in Andean records from Bolivia^22^, and Ecuador^20^ (Fig. 2). Beyond the overall glacial-interglacial temperature difference, our record reveals pronounced multi-centennial variability characterized by a two-step warming pattern, interrupted by a cooling phase of about 0.5 °C during the ACR. This cooling is also consistent with Andean glacier history^40^ and lake records from the Andes of Ecuador^20^.

The last warming phase features the fastest warming detected in the Rei do Mato speleothem. This rapid warming period (yellow rectangle in Fig. 2) was constrained by calculating linear regressions in our temperature record for several key intervals defined using breakpoint detection analysis (Supplementary Text 3, Supplementary Fig. 7, Supplementary Table 1). The good chronological control and sustained stalagmite growth rates during this interval support the robustness of the calculated trends (Supplementary Fig. 3b, d). The abrupt warming was characterized by a sharp 1.4 °C increase from 13.09 to 12.87 ka BP, at a rate of 0.64 °C/century (range: 0.05–1.23 °C/century). The YD warming marks a major shift in glacier dynamics in the tropical Andes, as glaciers exhibited a pronounced and widespread retreat during this interval, despite AMOC-driven increases in precipitation in the Peruvian and Bolivian Andes^17,40^. Following the YD, the retreat of most tropical glaciers continued into the early Holocene, when they reached their pre-industrial extent^17^.

The Rei do Mato record might be used as a benchmark for future projections of regional warming over central-eastern South America. In this context, it is interesting to compare the period of strongest deglacial warming in our record (upper range of 0.123 °C/decade) with the transient temperature response of 0.32 °C/decade (Fig. 1d) since 1980, driven by an increase in the global atmospheric CO_2_ concentration of 18.9 ppm/decade between 1980 and 2025^41^. This comparison shows how warming rates in recent decades far exceed the fastest rates we documented during the last deglaciation in this region. Given that the temperature response to a CO_2_ increase is transient and feedbacks have not yet reached an equilibrium with CO_2_ forcing, further increases in the rate of warming are expected in the coming decades until the climate system approaches equilibrium.

### Monsoon precipitation and temperature in central-eastern South America

In tropical regions, oxygen isotope ratios from rainfall and speleothem carbonates (δ^18^O_c_) primarily reflect precipitation variability linked to the amount and intensity of the convective processes within the regional hydrological cycle^42–46^. Ideally, when cave conditions remain approximately stable, the rainfall δ^18^O composition is transferred to speleothem carbonates through the drip water (δ^18^O_dw_), preserving the original climatic signal.

**Fig. 3:**
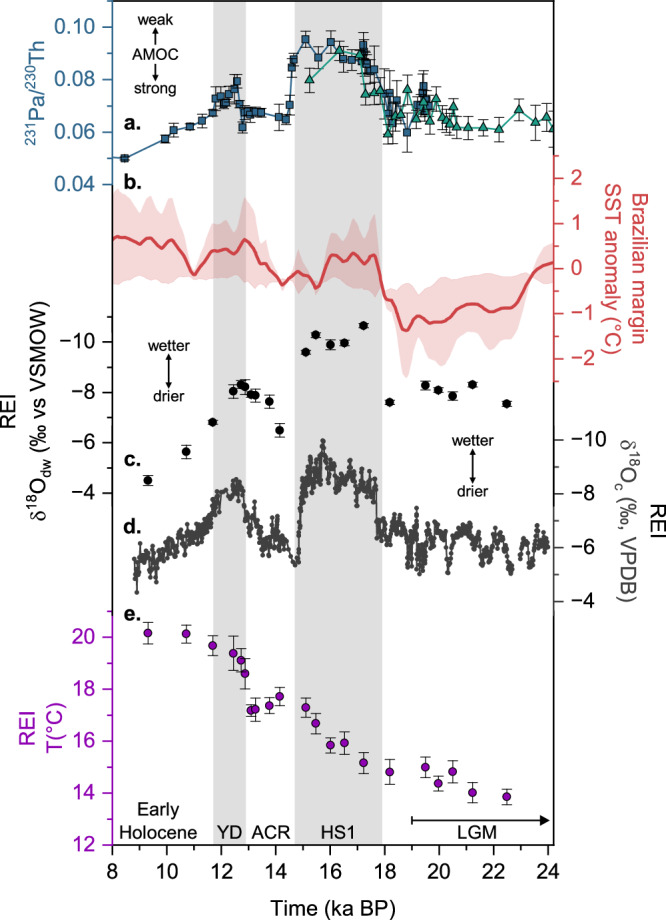
Comparison of Atlantic Meridional Overturning Circulation (AMOC) proxy records, sea surface temperature (SST), precipitation, and cave temperature evolution. **a** AMOC proxy ^231^Pa/^230^Th from ocean sediment core OCE326-GGC5^51^ (blue squares) and OCE326-GGC5^50^ (green triangles) with internal precision error bars (2 SD); **b** SST anomalies in the Brazilian margin (Brazil Current and North Brazil Current) (red line) with error of 1σ (red shading area)^57^; **c** Calculated drip water δ^18^O_dw_ based on δ^18^O_c_ and cave temperature, corrected for ice-volume related changes of mean ocean δ^18^O with 1 SD error bar; **d** δ^18^O_c_ and; **e** temperature reconstruction from Rei do Mato cave (this study). Gray vertical bars indicate Northern Hemisphere cooling episodes. YD Younger Dryas, HS1 Heinrich Stadial 1, ACR Antarctic cold reversal, LGM Last Glacial Maximum.

Calculated δ^18^O_dw_ values based on δ^18^O_c_, our cave temperature record, and corrected for the estimated 1 ‰ Glacial-Holocene change in mean seawater δ^18^O^47^ (Supplementary Text 3) reveal significant differences from the original δ^18^O_c_ record. During the glacial period, cave temperatures more than 5 °C cooler than today, combined with glacial enrichment of seawater δ^18^O, produced a significant increase in the δ^18^O_c_ record, independently of rainfall changes. However, our δ^18^O_dw_ record retains most of the millennial-scale variability of the δ^18^O_c_, especially around HS1. At the onset of the YD, however, the abrupt increase in δ^18^O_c_ that coincides with rapid warming is not reflected in the δ^18^O_dw_ record (Fig. 3). In general, when temperatures are known, calculated δ^18^O_dw_ can serve as a more direct indication of hydroclimate changes compared to classical δ^18^O_c_.

The δ^18^O_c_ and δ^18^O_dw_ records are consistent with the positive precipitation anomalies simulated by theTraCE21Kii experiment^48^ during HS1 and the YD, and with other speleothem records from eastern Brazil^5^ (Supplementary Fig. 8). Together, these records support the interpretation of intensified convective activity associated with the SAMS over central-eastern South America, in the context of a southward displacement of the ITCZ^5,49^, in response to an AMOC slowdown^50,51^ (Fig. 3). Overall, this AMOC-driven reorganization of tropical circulation represents the dominant mechanism of millennial-scale precipitation variability in the region^4,52,53^.

It is worth noting that one potential feedback mechanism linking temperature and precipitation involves cloud cover. Increased convective activity linked to increased cloud cover may lower temperatures by reducing incoming shortwave radiation. However, our δ^18^O_dw_ and cave temperature records show that changes in the hydrologic regime during millennial events were mostly decoupled from temperature variability, similar to findings from a cave in Borneo, where the same approach was used to derive deglacial temperatures and hydroclimate variability^29^ (Supplementary Fig. 9). Our records reveal that warming persisted after the abrupt precipitation events had ended. Moreover, the abrupt temperature rise at the onset of the YD occurred during a phase of high precipitation, as indicated by lower δ^18^O_c_ and δ^18^O_dw_ values of the same speleothem. Thus, over our study site, the cloud feedback mechanism does not seem to have significantly affected the long-term deglacial warming trend during this period.

### Temperature response to CO_2_ and AMOC forcings

The cave temperature reconstruction is overall consistent in magnitude and timing with the transient model simulation of surface temperature from the TraCE21Kii experiment^48^, performed with the Community Climate System Model version 3 (CCSM3)^54,55^. This good correspondence allows us to investigate the model simulations more closely to determine the drivers behind the reconstructed temperature evolution.

**Fig. 4:**
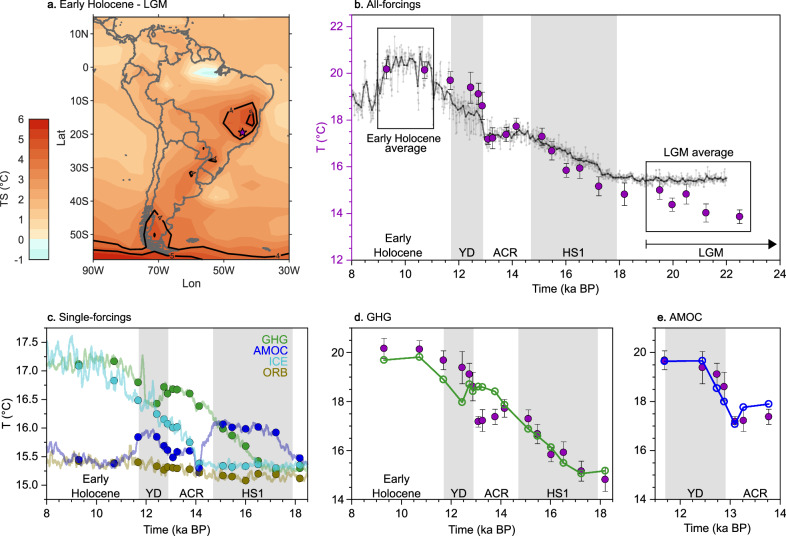
Comparison between temperature reconstructed from Rei do Mato and surface temperature from TraCE21Kii experiment^48^ and stepwise regression with single forcings. **a** Early Holocene average (9–11 ka BP) minus Last Glacial Maximum average (19–22 ka BP) surface temperature difference based on TraCE21Kii experiment^48^. Black contours highlight regions with a temperature difference of more than 4 °C; **b** Temperature evolution from microthermometry (purple symbols) with 2SEM error bars, and TraCE21kii decadal averages (thin gray line) and 100-year moving-average (bold black line) at Rei do Mato. Boxes indicate the time periods used to calculate the temperature difference shown in panel (**a**); **c** Color lines represent 100-year moving-average surface temperatures at our cave site, as simulated by TraCE21kii single-forcing experiments: Greenhouse gases (GHG), ice (ICE), orbital (ORB) and AMOC forcing. Symbols indicate the values used for the stepwise regression. **d** Deglacial GHG single-forcing response and; **e** AMOC single-forcing response for the ACR-YD interval compared with our proxy-based record, where the estimated value represents the strength of the effect of the predictor on the response (Supplementary Text 3). Gray vertical bars indicate Northern Hemisphere cooling episodes. YD Younger Dryas, HS1 Heinrich Stadial 1, ACR Antarctic cold reversal, LGM Last Glacial Maximum, AMOC Atlantic Meridional Overturning Circulation.

The TraCE21Kii experiment combines greenhouse gases (GHG), ice sheet (ICE) and orbital (ORB) forcings, and AMOC-induced changes in ocean heat transport (AMOC) (Fig. 4a, b). Although the latter is often referred to as freshwater forcing (FWF), it should be noted that in the TraCE21Kii simulations, this is not intended as a realistic reconstruction of meltwater flux, which is known to be physically inconsistent with certain ice-sheet reconstructions (e.g., ref. ^56^). Instead, the FWF in TraCE21Kii serves as a numerical driver to prescribe an AMOC state that matches paleoclimate records (Supplementary Fig. 10). Our comparison, therefore, evaluates the temperature response to these prescribed AMOC-driven changes in heat transport.

To assess the role of individual forcings in driving temperature variability over the region, we conducted a stepwise regression analysis using our reconstructed surface temperature data alongside single-forcing surface temperature experiments with GHG, AMOC, ICE, and ORB (Fig. 4c–e, Supplementary Text 3). The analysis supports the primary effect of GHG on the long-term deglacial temperature evolution (Fig. 4d), while AMOC forcing accounts for millennial variability, most evident at the onset of the YD (Fig. 4e). Notably, this abrupt warming is one of the most striking features both in our record and in the TraCE21Kii transient simulation (Fig. 4b).

The AMOC forcing likely contributes to the initial deglacial warming in central-eastern South America during HS1. As shown by proxy-based sea surface temperature (SST) records from the South Atlantic^57^, the AMOC weakening leads to heat accumulation in this ocean basin (Fig. 3c). A local rapid SST warming was detected in the South Atlantic off southeastern Brazil at 21.5 °S^58^, although the latter is not reflected in other AMOC records. This AMOC-driven excess of energy contributed significantly to continental warming^59^, and presumably also during the YD. Yet, there are currently no proxy records documenting a rapid regional SST response to AMOC forcing in the Brazilian margin during that time.

Overall, we show that the Trace21Kii experiment provides an accurate picture of temperature evolution during the deglacial period in central-eastern South America, warming more than the rest of the continent through the deglacial period, and reproducing many key features associated with millennial-scale variability (Fig. 4a). The all-forcings experiment (Fig. 4b) successfully reproduces the cooling during the ACR within uncertainties, likely reflecting the influence of reduced AMOC forcing, which is supported by the consistency between the AMOC single forcing experiment and our cave record (Fig. 4c). Nonetheless, the Rei do Mato record also reveals an interval of non-linear regional temperature response to GHG forcing, observed during the ACR-YD transition. Given the robust nature of our cave record, we provide a crucial benchmark for understanding temperature evolution in central-eastern South America. This study highlights the need for additional precise paleotemperature records from other regions where different climate factors may be at play.

## Methods

### Nucleation-assisted microthermometry

Microthermometric measurements were performed on a Linkam THMSG600 heating/cooling stage attached to an Olympus BX51 microscope equipped with a 100× long-working-distance objective (Olympus LMPLFLN 100×/0.8) and a Leica DFC350 FX digital camera for visual observation. The heating/cooling stage was calibrated with synthetic fluid inclusion standards (pure H_2_O, H_2_O-CO_2,_ and H_2_O-NaCl) using the triple point of water (0.0 °C), the critical temperature of CO_2_ (31.4 °C), and the eutectic temperature (−21.2 °C), respectively. The temperature accuracy of the Linkam stage was ±0.1 °C. An amplified Ti:Sapphire femtosecond laser (CPA-2101, Clark-MXR, Inc) that provides single ultra-short laser pulses is coupled into the microscope light path and focused on the sample through the 100× objective to stimulate vapor bubble nucleation in metastable liquid fluid inclusions. For a detailed description of the analytical setup, see ref. ^30^.

To measure liquid-vapor homogenization temperatures in single-phase liquid fluid inclusions, the sample needs to be cooled to a couple of degrees below the expected homogenization temperature to transfer the enclosed water from a stable into a metastable liquid state. Subsequently, vapor bubble nucleation can be triggered by a single ultra-short laser pulse focused through the microscope objective onto the inclusion^28^. After bubble nucleation, the inclusion is in a stable liquid-vapor equilibrium state. Upon subsequent heating, the liquid phase expands until the vapor bubble collapses, and the inclusion homogenizes into a stable liquid state again. The temperature at which the vapor bubble collapses is called the observed homogenization temperature (T_h(obs)_). Using additional measurements of the vapor bubble radius at a known temperature, the effect of surface tension on T_h(obs)_ can be calculated using a thermodynamic model^39^. The resulting corrected homogenization temperature is called T_h∞_ and is assumed to directly relate to the temperature at which the inclusion sealed off from the cave environment.

Each temperature data point in our record represents the average T_h∞_ of about 40 individual fluid inclusions measured along the selected growth bands. The T_h∞_ values typically exhibit a Gaussian distribution, and the mean is considered the best estimate of the formation temperature of the corresponding growth band (Supplementary Fig. 4). The temperature distributions were statistically analyzed to remove outliers using a conservative 4 Median Absolute Deviation (4MAD) criterion. A total of 857 fluid inclusions were analyzed, of which less than 1.2% were identified as outliers. We did not measure T_h(obs)_ above a selected threshold of 27 °C to avoid high fluid overpressure and stretching of other inclusions with lower T_h(obs)_ in the sample. Inclusions that showed increasing T_h(obs)_ values across replicate measurements or did not homogenize below the threshold temperature of 27 °C were not included in the statistical analysis due to stretching during measurements and sample preparation, respectively.

### Stable isotope measurements in carbonate

Sample preparation and measurements were performed at the Stable Isotope Laboratory of the Geoscience Institute of the University of Sao Paulo (IGc-USP). The δ^18^O_c_ and δ^13^C_c_ transect was measured on Slab 1 (Supplementary Text 2 and Supplementary Fig. 3). Sample aliquots of 100 to 200 μg were prepared with a microsampler (Sherline, model 5400) with a sampling resolution of 0.4 mm.

Analyses were performed on a Thermo Finnigan Delta Plus Advantage IRMS fitted with a Finnigan Gas-Bench II automatic sample preparation system. Isotope values are reported as δ^13^C_c_ and δ^18^O_c_ in ‰ relative to VPDB, with standard reproducibility (1σ) better than 0.1 ‰ in both cases. The final isotope profile after the cleanup of erroneous values comprises 933 data points for δ^18^O_c_ and 933 for δ^13^C_c_.

### Chronology

The speleothem chronologies were obtained by U-series disequilibrium dating (U-Th method) at the Institute of Global Environmental Change at Xi’an Jiaotong University (China) and at the Geochronology Laboratory of the University of Minnesota (USA). An MC-ICP-MS (Multi-collector Inductively Coupled Plasma Mass Spectrometry) Finnigan Elements and Finnigan Neptune Type were used to analyze the sample solutions following the procedures established by ref. ^60^. The age calculations were performed using measured isotopic ratios and correction factors to eliminate the effects of contamination by detrital ^232^Th, following refs. ^61,62^.

Aliquots of ~0.1 g of carbonate powder (CaCO_3(s)_) were drilled using a Dremel rotary tool and stored in glass vials. The samples were extracted at different depths along the growth axis of each stalagmite, from top to bottom. Samples were taken along a single layer. Layers with potential post-depositional alterations or detrital material (mud layers) that could be a source of ^232^Th from the clay minerals were avoided, as these would lead to inaccurate ages.

The age model was constructed from the U-series ages using the StalAge software, which allows identification of outliers, age inversions, hiatuses, and large changes in growth rate^63^. Age models and corresponding 95% confidence limits are obtained via Monte Carlo simulation by fitting ensembles of straight lines to subsets of age data^63^.

## Supplementary information

Supplementary Information

Description of Additional Supplementary Files

Supplementary Code 1

Transparent Peer Review file

## Source data

Source Data

## Data Availability

The proxy and monitoring records generated in this study are provided in the Source Data file and deposited in the PANGAEA database [10.1594/PANGAEA.995231, 10.1594/PANGAEA.995230, 10.1594/PANGAEA.995223, 10.1594/PANGAEA.995229, 10.1594/PANGAEA.995228]. Global Precipitation Measurement Mission [10.5067/GPM/IMERG/3B-MONTH/07]. ERA 5 T2m reanalysis [Copernicus Climate Change Service (2022): ERA5-Land monthly averaged data from 1950 to present. Copernicus Climate Change Service (C3S) Climate Data Store (CDS). 10.24381/cds.68d2bb30 (Accessed on 07-JUL-2025)]. Lapa Sem Fim [https://doi.pangaea.de/10.1594/PANGAEA.847065]. Paixao [https://doi.pangaea.de/10.1594/PANGAEA.847072]. Noble gas temperature record [10.1126/science.269.5222.379]. Core GeoB6211-2 [https://doi.pangaea.de/10.1594/PANGAEA.847352]. Lake Llaviucu [https://www.ncei.noaa.gov/access/paleo-search/study/39579]. Glacier equilibrium lines [10.1016/j.quascirev.2020.106542]. CO_2_ [10.25921/n8y4-bp27]. EDC [10.1594/PANGAEA.934094]. SST [10.1594/PANGAEA.946131]. AMOC1 [10.25921/abs9-9f10]. AMOC2 [10.1038/nature14059]. TraCE21Kii [https://trace-21k.nelson.wisc.edu/portal.html]. Source data are provided with this paper.

## Code availability

The “Breakpoint detection” and “Trend-fitting” analyses were performed using MATLAB (R2023a). The MATLAB codes used are provided in Supplementary Code 1.
