# Supplementary material for: Rapid warming in South America during the last deglaciation
Source: Nat Commun. 2026 Jun 20;17:8068. doi: 10.1038/s41467-026-74093-x (PMC13454564; doi:10.1038/s41467-026-74093-x)
Supplement: Supplementary file 1 [file 41467_2026_74093_MOESM2_ESM.pdf]

Description of Additional Supplementary Files

File name: Supplementary Code 1

Description:

This collection of MATLAB code is a supplement to the paper: Ampuero et al., Rapid Warming in South America during the Last Deglaciation (under revision)

The code performs the "breakpoint detection" and "trend-fitting" analysis as described in that work.

First, the "breakpoints" are identified and then the "trend-fitting" is performed in each segment following a Monte Carlo approach.

To run the code, the user should put the name of .csv file in Line 27. The file should include three columns (first: time [years]; Second: temperature [°C]; Third: uncertainty [2SEM]) with no title.

The .csv file should be put in the same folder of this code

The user can set the maximum number of breakpoints (cp_max) in Line 23 and the number of iterations (n_iter) in Line 25.
