# Supplementary material for: Rapid warming in South America during the last deglaciation
Source: Nat Commun. 2026 Jun 20;17:8068. doi: 10.1038/s41467-026-74093-x (PMC13454564; doi:10.1038/s41467-026-74093-x)
Supplement: Supplementary file 2 [file 41467_2026_74093_MOESM4_ESM.pdf]

Peer Review File

Rapid Warming in South America during the Last Deglaciation

Corresponding Author: Dr Angela Ampuero Grandez

This file contains all reviewer reports in order by version, followed by all author rebuttals in order by version.

Version 0:

Reviewer comments:

Reviewer #1

(Remarks to the Author)
Rapid warming events in tropical South America during the last deglacial

The authors present a new speleothem record from the southeastern Neotropics, providing both a temperature and a precipitation record of the LGM to early mid Holocene. This is an important new contribution as there are so few empirical records of temperature variation in this period. I found this manuscript to be well-written and have relatively minor comments.

I found it surprising that van Breukelen et al. (2008) were not cited as their paper seems to me to be the most similar analysis on inferring temperature from fluid inclusions in a speleothem yet published from South America.

I realize that the authors are looking mainly at other isotopic or noble gas analyses to make comparisons, but the first serious evidence for LGM cooling (4oC) in lowland South America came from macrofossils (Liu and Colinvaux 1985) and from pollen (5oC) (Bush et al. 1990 and Colinvaux et al. 1996). Similarly, many papers from Ledru, De Oliveira and others have described the cooling of caatinga and cerrado systems and the transition to modern warmth and the impact of H-1. It seems something of a wasted opportunity not to join these dots and point out that the new isotopic data are entirely consistent with vegetation reconstructions.

I would like to see the chronology- rbacon model or whatever- in the main text.

L. 56 Tenet not tenant
L. 124 to the cave's
Fig. 1 increase the size of the lettering.
L. 169 Is there a modern temperature from current drip water or speleothem as opposed to an instrumental value, i.e. trying to determine if there is any offset?
L. 180 There appears to be a strong ACR response, which is also seen in other regional records, yet not much is said about it.
Fig. 3 Help the reader by adding arrows indicating wetter or drier.
L. 346 delete 'In order"

References
van Breukelen, M.R., H.B. Vonhof, J.C. Hellstrom, W.C.G. Wester, and D. Kroon. 2008. Fossil dripwater in stalagmites reveals Holocene temperature and rainfall variation in Amazonia. Earth and Planetary Science Letters, 275, 54-60

M. B. Bush, P. A. Colinvaux, M. C. Wiemann, D. R. Piperno and K.-B. Liu. Late Pleistocene temperature depression and vegetation change in Ecuadorian Amazonia. Quaternary Research 1990 Vol. 34 Pages 330-345

P. A. Colinvaux, P. E. De Oliveira, J. E. Moreno, M. C. Miller and M. B. Bush. A long pollen record from lowland Amazonia: forest and cooling in glacial times. Science, Vol. 274 Issue 5284 Pages 85-88.

M.-P. Ledru, P. Mourguiart and C. Riccomini

Related changes in biodiversity, insolation and climate in the Atlantic rainforest since the last interglacial. Palaeogeography, Palaeoclimatology, Palaeoecology 2009 Vol. 271 Issue 1 Pages 140-152.

Liu K.-B. and P. A. Colinvaux. Forest changes in the Amazon Basin during the last glacial maximum. Nature 1985 Vol. 318 Pages 556-557.

Reviewer #2

(Remarks to the Author)
This is an interesting study of temperature changes in the South American tropics coincident with periods of rapid climatic change during the last deglaciation. The method used to reconstruct paleotemperatures (nucleation-assisted fluid inclusion microthermometry) is rapidly gaining traction and appears to provide robust and precise values. The dating of the stalagmite appears solid, as evidenced, in part, by only small changes between ages prior to and following the detrital Th correction, the small errors, and the stratigraphic consistency. The other paleotemperature reconstructions from the region allow important points of comparison, as does the CMIP6 downscaled climate model simulation.

As I read it, the most important goal of this research is to allow a point of comparison (periods of rapid warming during the last deglaciation) for projected future climate changes. To me, this gets a little lost in the weeds, first because the writing could be tightened, and second because climate model boundary conditions are not sufficiently defined. The authors do a good job of explaining that temperature change during the YD and HS1 was not driven solely by GHG but also by AMOC, cloud cover, etc. But if we are comparing past warming to projected future warming, why not also quantify changes in GHG? They are rising much faster today than they did during the last deglaciation. This should be quantified. Likewise, why focus solely on projections of climate? Why not using well documented warming of the last several decades (presented in Fig 1d) as a point of comparison? The results presented in this study ring an alarm bell. This area of South America is warming faster today (and will continue to do so even under optimistic emissions scenarios) than it did during periods of massive reorganizations in the circulations of the oceans and atmosphere.

9 – since you are linking elevated CO_2/temperatures during deglaciation to elevated CO_2/temperatures today and in the future, perhaps quantify the magnitude and rate of CO_2 increase from the LGM to early Holocene and then provide note that it is exceeded by the magnitude and rate of anthropogenic emissions over the last century. You must be careful not to ascribe all of the warming to CO_2 because, as the authors note, complex feedbacks, many of which are difficult to capture in climate model simulations, also play important roles. But CO_2 certainly plays a role so quantifying it, while also providing these caveats, seems like an important component of the discussion.

13 – is it a lack or a paucity? Perhaps also precise temperature records? Also, I'm not sure what "in-situ" refers to here.

14 – delete comma

15 – delete "21st century"

24 – I know it's redundant, but perhaps write "projections of future long-term"

33 – delete "the" before Heinrich?

34 – replace dashes on this line with commas

43 – delete "in South America"

51 – "ice sheet/albedo"?

62 – delete "at most on multi-centennial timescales"

65 – "bear", not "bare"

65 – perhaps just "often bear uncertainties exceeding 2˚C"

89 – have you compared historical temperature data from the cave site (or a neighboring town/weather station) to those across the broader region? Do they move in tandem? It would help to prove that there are no microclimatic effects impacting the site.

92 – "By applying…"

102 – what is the size of the opening? Which direction does the opening face? Are there bats in the cave today?

114 – "21.3 ˚C"

115 – delete “for 19 months”

117 – is the range in temperature?

117 – delete “high”

119 – do drips (and stalagmite deposition) continue throughout the dry season? This is important to know because if not, then your reconstruction is biased toward warmer, dry season cave temperatures.

121– in a shallow cave, could evaporative cooling near the entrance play a role in determining cave temperature? Under periods of changing monsoon rainfall, this could potentially influence cave temperature.

Where in the cave were the stalagmites collected?

146, “(2011), which”

155 – I'm unclear what a growth band is in this context

169 – It would help to be able to start by reporting the modern cave temperature as determined through this microthermometry method and comparing it to the measured temp. Are there actively growing (or quite young) samples that have been analyzed?

172 – noble gas-derived

178 – I'm not sure of how the specific conventions of Nature Communications, but do you need to define “ka B.P.” here?

191 – it would be helpful to provide a few specific examples of glaciers that retreated rapidly at this time. Perhaps plot them on the map in Figure 1?

192 – perhaps “where AMOC-driven precipitation increases offset melting, leading to glacial advances”

196 – 23rd century?

197 – can you contextualize the SSP2-4.5 scenario by providing the magnitude of increase in atmospheric GHG: e.g., 1 ppm CO2 equivalent per year?

198 – ditto for SSP5-8.5 scenario

200 – “projections from phase 6”

233 – I'm unclear what is meant by “positive bias” here.

237 – lower sea levels mean substantially more exposed continental shelf on the eastern coast of South America. This could have impacted precipitation (and thus stalagmite) d18O values further inland.

240 – the punchline of this paragraph is a bit lost on me

277 – “2022), which”

280 – “the reconstructed temperature”

288 – here you state that the transient model simulations do a good job of capturing the temperature variability reconstructed by the stalagmites. However, in the abstract (line 22), you appear to make the opposite argument.

291 – proxy-based

300 – “the temperature evolution”

399 – replace wrong with inaccurate

400 – “The age model was constructed from the U-series ages using the StalAge software, which allows identification of outliers, …”

Figure 1 – since panel b and d contain purple data points representing temperature, perhaps use a different color for the bars denoting rainfall.

Supplemental Material
7 – tourist cave

9 – what are the dimensions of the cave (height, width) and of the opening? Include a cave map. (coming back to this after reading further down the SM: I suggest moving this map up to the intro of the SM).

13 – tourist

33 – delete second comma

38 – “19 months of”

43 – why the lag?

68 – delete comma

105 – delete comma

168 – “from Slab 2. (b)…”

The data table showing U/Th isotope ratios contains some font mistakes in the headings.

Reviewer #3

(Remarks to the Author)
The manuscript presents a new speleothem record from a cave in south-eastern Brazil. Speleothems are now widely recognized as valuable paleoclimate archives, as they have the potential to provide unbiased and accurately dated records of changes in annual mean surface air temperature and the hydrological cycle. As South America is not yet well covered by such records, this new record is a valuable addition to the study of regional climate change during the last glacial termination. However, regardless of how good an individual paleoclimate record is, it is just one record and is likely to show local climate variability. Therefore, it is surprising that the authors based their very bold statements, such as claiming to have found a “significant nonlinear response of the climate system to radiative and freshwater flux forcings”, on a single record. It is unclear what the authors mean by “response of the climate system” (annual mean temperature?) and how they concluded that the response to radiative forcing (from greenhouse gases?) is strongly nonlinear. The issue of the climate response to freshwater forcing is discussed below.

My second problem with the manuscript under consideration is that, unlike the authors, I cannot see two rapid warming events in their data. There is no doubt that the reconstructed temperature rise at 13 ka is abrupt, if not nearly instantaneous. However, the reported abrupt warming between 16 ka and 15 ka is attributed solely to a single data point at around 16 ka. If this outlier is excluded, the best statistical model for the period between 18 ka and 14 ka is a linear temperature rise, which is fully consistent with a similar rise in CO_2 concentration (see also their Figure S7). A small temperature decline during the ACR period can also be easily explained by the stabilization of CO_2 during this time, as well as the “bipolar seesaw”. Thus, the only mysterious aspect of the new record is the abrupt warming at the beginning of the Younger Dryas, with temperatures rising by around 2°C in just one hundred years. Such abrupt and strong warming cannot be explained by greenhouse gases or the classical bipolar seesaw, given the timescale of the Southern Hemisphere response. Therefore, it must be a very localized effect. The cited paper by Pedro et al. (2018) shows the appearance of a very local warming spot just 100 years after the AMOC shut down, not far from the location of the cave. However, this result is unlikely to be robust, as it is not evident in other simulations of AMOC shutdown. In any case, regardless of whether this abrupt warming is real, it cannot be considered a large-scale phenomenon. Furthermore, it is unclear what the authors meant by “tipping point” (line 190) in the context of their study.

Concerning the climate response to the freshwater flux (FWF): When comparing the results of the TraCE21Kii experiments performed with the CCSM3 model (mistakenly referred to as the “TraCE21Kii model” by the authors), it should be noted that the freshwater forcing used in these experiments (as well as in the initial TraCE21K experiment by Liu et al. 2009) is completely inconsistent (in fact, inverted) with the global ice sheet reconstructions, the fact recognized by the paleoclimate modelling community already for a long time. Real reconstructions of the freshwater flux into the North Atlantic (see, for example, Kapsch et al., 2022, Figure 1), derived from ice sheet evolution reconstructions (Tarasov et al. 2012; Peltier et al. 2015), show a maximum in FWF during the warm Bolling interval and after the Younger Dryas, with a rather small FWF during the Heinrich 1 and Younger Dryas periods. This is the opposite of the FWF scenario used in the TraCE21Kii experiment. Thus, when the authors compare their results with those of TraCE21Kii, they compare real climate change with the FWF scenario designed by Liu et al. (2009) and He & Clark (2022) solely to achieve the best fit between their climate model results and paleoclimate reconstructions. As the actual FWF during the last glacial termination differs significantly from that used in TraCE21Kii, the discussion of the role of freshwater flux in climate change during past glacial terminations (pages 14 and 15) is fundamentally flawed.

Kapsch, M.-L., Mikolajewicz, U., Ziemen, F., & Schannwell, C. (2022). Ocean response in transient simulations of the last deglaciation dominated by underlying ice-sheet reconstruction and method of meltwater distribution. Geophysical Research Letters, 49, e2021GL096767.
Liu, Z., Otto-Bliesner, B.L., He, F., Brady, E.C., Tomas, R., Clark, P.U., Carlson, A.E., Lynch-Stieglitz, J., Curry, W., Brook, E. and Erickson, D., (2009). Transient simulation of last deglaciation with a new mechanism for Bølling-Allerød warming. Science, 325, 310-314.

Peltier, W. R., Argus, D. F., & Drummond, R. (2015). Space geodesy constrains ice age terminal deglaciation: The global ICE-6G_C (VM5a) model. Journal of Geophysical Research: Solid Earth, 120(1), 450–487.
Tarasov, L., Dyke, A. S., Neal, R. M., & Peltier, W. (2012). A data-calibrated distribution of deglacial chronologies for the North American ice complex from glaciological modeling. Earth and Planetary Science Letters, 315–316, 30–40.

Version 1:

Reviewer comments:

Reviewer #1

(Remarks to the Author)
I believe that the authors have done a good job of responding to referees criticisms, and this paper while being inconsistent in its findings with other that show more gradual warming will trigger further inquiry.

I suggest the authors tweak the sentence on line 82 where they seem to suggest that it is odd that the Fuquene record should be responding to Greenland. Overall the dominant pacing of long records north of about 11oS, i.e. Lakes Junin and Fuquene and speleothems Santiago cave and Cueva del Diamante, all of which show AMOC forcing and are therefore in synch with Greenland rather than Antartica. Sites south of this line (which probably shifts between 9-11oS according to glacial stage) are more likely to show Antarctic forcing (Bradbury 2001)

Full and late glacial lake records along the PEP1 transect: Their role in developing interhemispheric paleoclimate interactions

J. P. Bradbury, M. Grosjean, S. Stine and F. Sylvestre

In: Interhemispheric Climate Linkages, edited by V. Markgraf

Academic Press 2001

Reviewer #2

(Remarks to the Author)
I reviewed an earlier version of this manuscript and expressed several concerns about the arguments being made. The authors are now to be commended for addressing these concerns in detail. The paper now reads quite well, and I feel it is ready for publication.

Reviewer #3

(Remarks to the Author)
I am fully satisfied with the author's response to my criticism, and I would recommend the manuscript for publication in Nature Communications.

Open Access This Peer Review File is licensed under a Creative Commons Attribution 4.0 International License, which permits use, sharing, adaptation, distribution and reproduction in any medium or format, as long as you give appropriate credit to the original author(s) and the source, provide a link to the Creative Commons license, and indicate if changes were made.
In cases where reviewers are anonymous, credit should be given to 'Anonymous Referee' and the source.
The images or other third party material in this Peer Review File are included in the article's Creative Commons license, unless indicated otherwise in a credit line to the material. If material is not included in the article's Creative Commons license and your intended use is not permitted by statutory regulation or exceeds the permitted use, you will need to obtain permission directly from the copyright holder.
To view a copy of this license, visit https://creativecommons.org/licenses/by/4.0/

Response to reviewer's comments

REVIEWERS' COMMENTS:

Reviewer #1 (Remarks to the Author):

The authors present a new speleothem record from the southeastern Neotropics, providing both a temperature and a precipitation record of the LGM to early mid Holocene. This is an important new contribution as there are so few empirical records of temperature variation in this period. I found this manuscript to be well-written and have relatively minor comments.

I found it surprising that van Breukelen et al. (2008) were not cited as their paper seems to me to be the most similar analysis on inferring temperature from fluid inclusions in a speleothem yet published from South America.

Reply: We thank the reviewer for this observation. Van Breukelen et al. (2008) are indeed the pioneers in reconstructing temperatures using the fluid inclusion-calcite thermometer in South America. Yet, we decided not to cite it because it spans a different period (it covers only the Holocene), and the fluid inclusion-calcite thermometer and microthermometry are completely independent methods that rely on different physical principles.

I realize that the authors are looking mainly at other isotopic or noble gas analyses to make comparisons, but the first serious evidence for LGM cooling (4oC) in lowland South America came from macrofossils (Liu and Colinvaux 1985) and from pollen (5oC) (Bush et al. 1990 and Colinvaux et al. 1996). Similarly, many papers from Ledru, De Oliveira and others have described the cooling of caatinga and cerrado systems and the transition to modern warmth and the impact of H-1. It seems something of a wasted opportunity not to join these dots and point out that the new isotopic data are entirely consistent with vegetation reconstructions.

Reply: We thank the reviewer for drawing attention to these important papers, which we have now incorporated into the main manuscript. We added new text in the Introduction (lines 72 and 75) that cites the temperature assessment derived from the Amazonian pollen record.

Lines 72-75: "Pollen records from western Amazon points that during the last glacial period, temperatures were 4.5 to 6ºC cooler than today, allowing cold-adapted Andean species to thrive in the lowland despite the continued presence of the rainforest (Liu, 1985; Colinvaux, 1996)."

References:

P. A. Colinvaux, P. E. De Oliveira, J. E. Moreno, M. C. Miller and M. B. Bush. A long pollen record from lowland Amazonia: forest and cooling in glacial times. Science, Vol. 274 Issue 5284 Pages 85-88.

Liu K.-B. and P. A. Colinvaux. Forest changes in the Amazon Basin during the last glacial maximum. Nature 1985 Vol. 318 Pages 556-557.

I would like to see the chronology- rbacon model or whatever- in the main text.

Reply: We appreciate the reviewer's suggestion. However, given the limited space, we have chosen to present the StalAge-derived age model in the Supplementary Figure 3 and the methods are described in the main manuscript, in the lines 402-422 (Chronology). The option we adopted follows the standard practice in speleothem publications.

L. 56 Tenet not tenant

Replaced "*tenant*" with "*tenet*" (line 55).

L. 124 to the cave's

Replaced *"…cold-trap behavior related to cave's ventilation regime*" with "*cold-trap behavior related to the cave ventilation regime*" (lines 129).

Fig. 1 increase the size of the lettering.

Lettering increased.

Original:

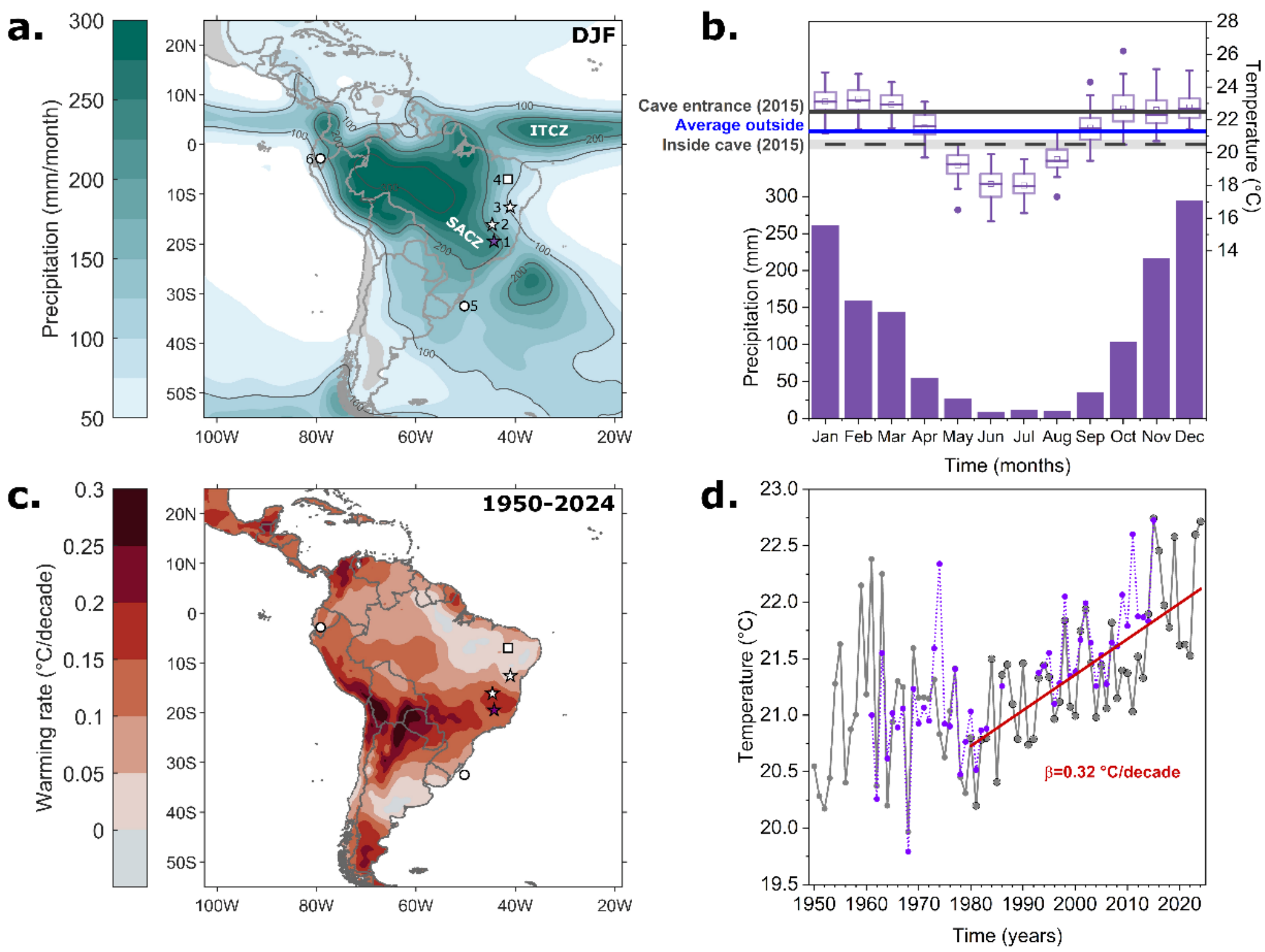

Modified:

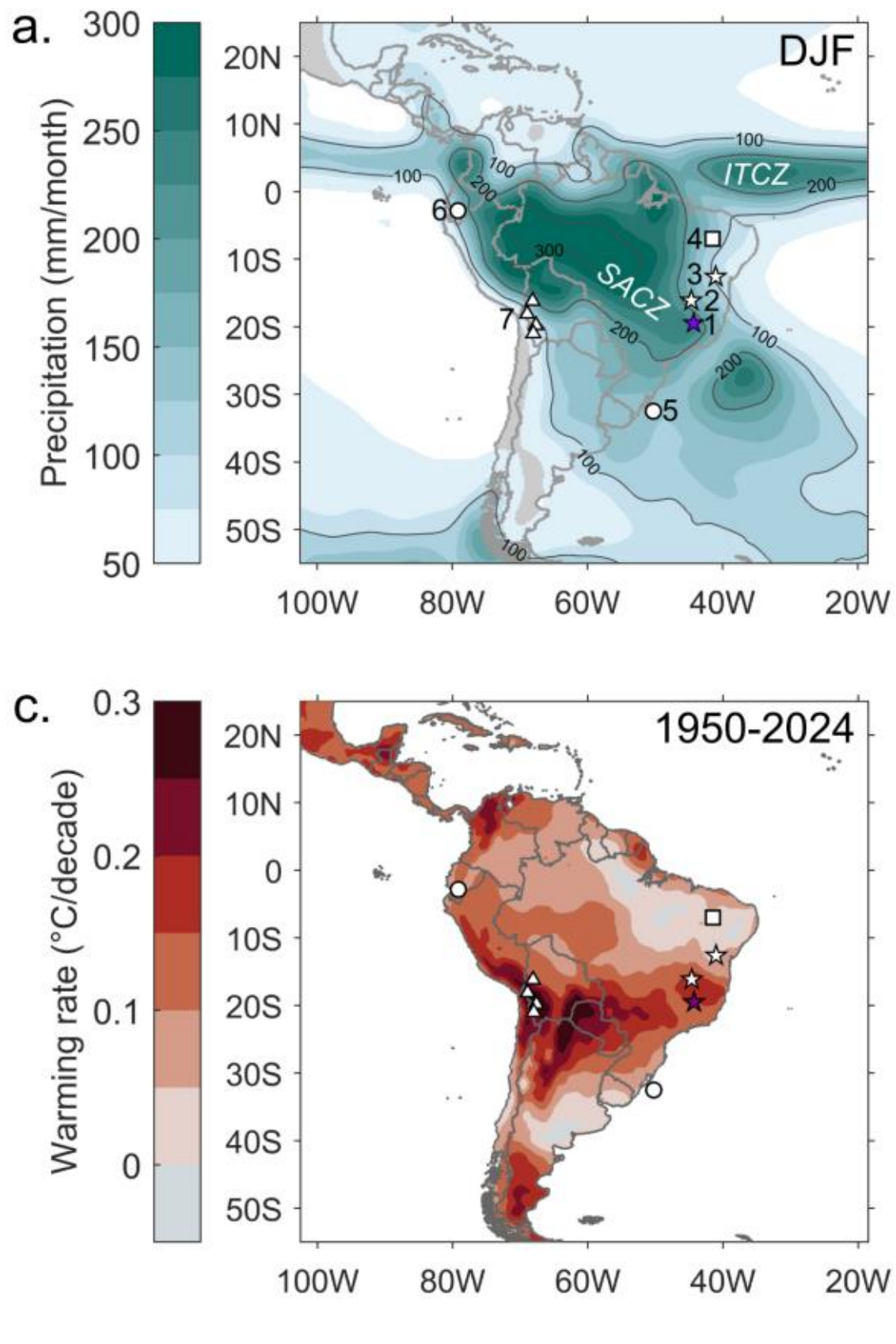

L. 169 Is there a modern temperature from current drip water or speleothem as opposed to an instrumental value, i.e. trying to determine if there is any offset?

Reply: This is a very important observation. To address this, we performed additional measurements at the top of a modern stalagmite (REI 7-7), which yielded values consistent with the observed cave temperature (Figure 2). Additional information about stalagmite REI 7-7 was also included in (lines 163-167) and in the supplementary figure S5.

lines 163-167: "*Additionally, two temperatures measured at the top of a modern stalagmite (REI 7-7) collected near our deglacial record (REI7-2), match observed temperatures during 2015, supporting the accuracy of the microthermometry temperatures at Rei do Mato cave (Fig. 2d and Supplementary Fig. 5).*"

Original:

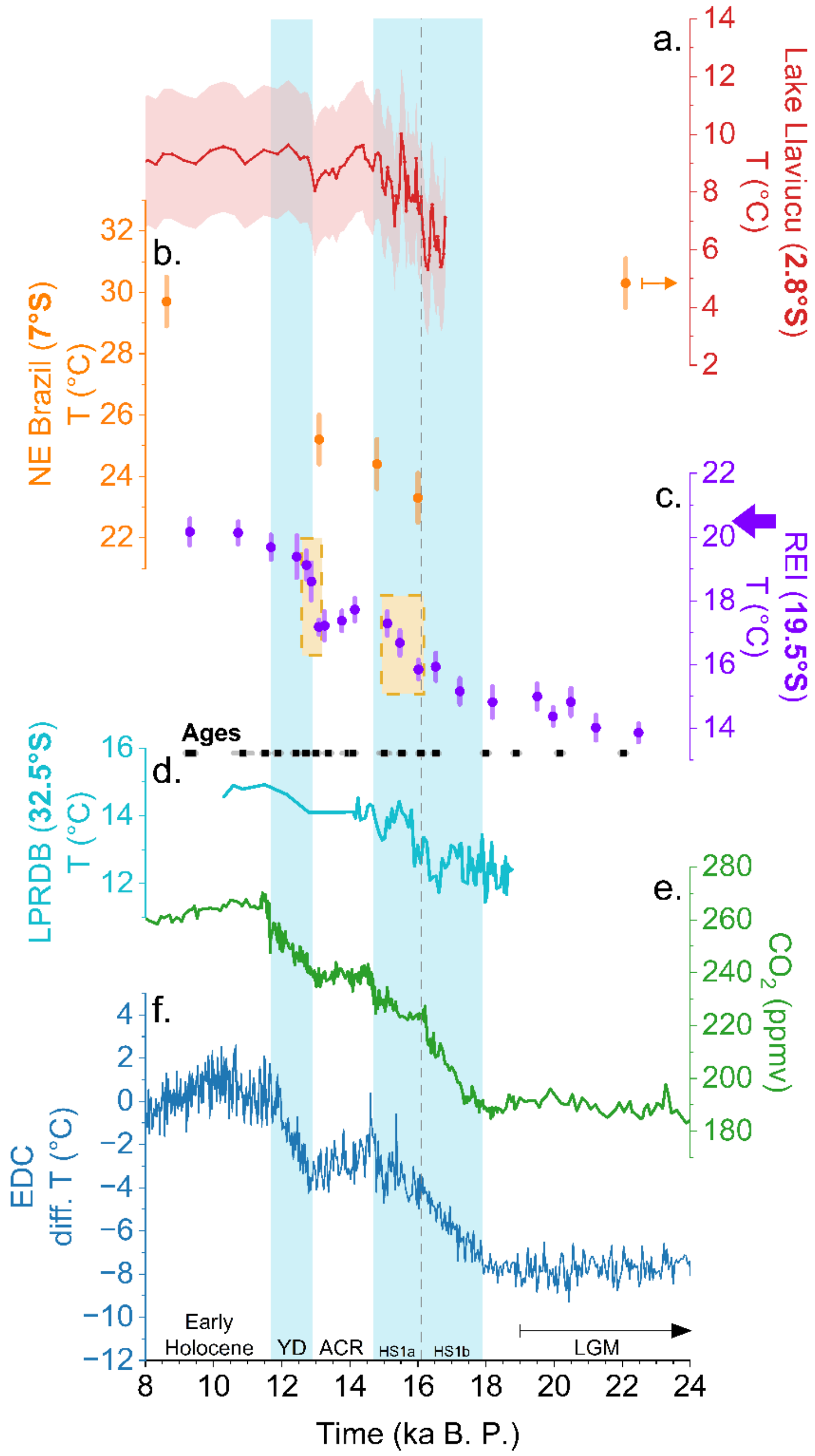
a.
Lake Llaviucu (2.8°S)
T (°C)
b.
NE Brazil (7°S)
T (°C)
c.
REI (19.5°S)
T (°C)
Ages
d.
LPRDB (32.5°S)
T (°C)
e.
CO_2 (ppmv)
f.
EDC
diff. T (°C)
Early Holocene
YD
ACR
HS1a
HS1b
LGM
Time (ka B. P.)

Modified:

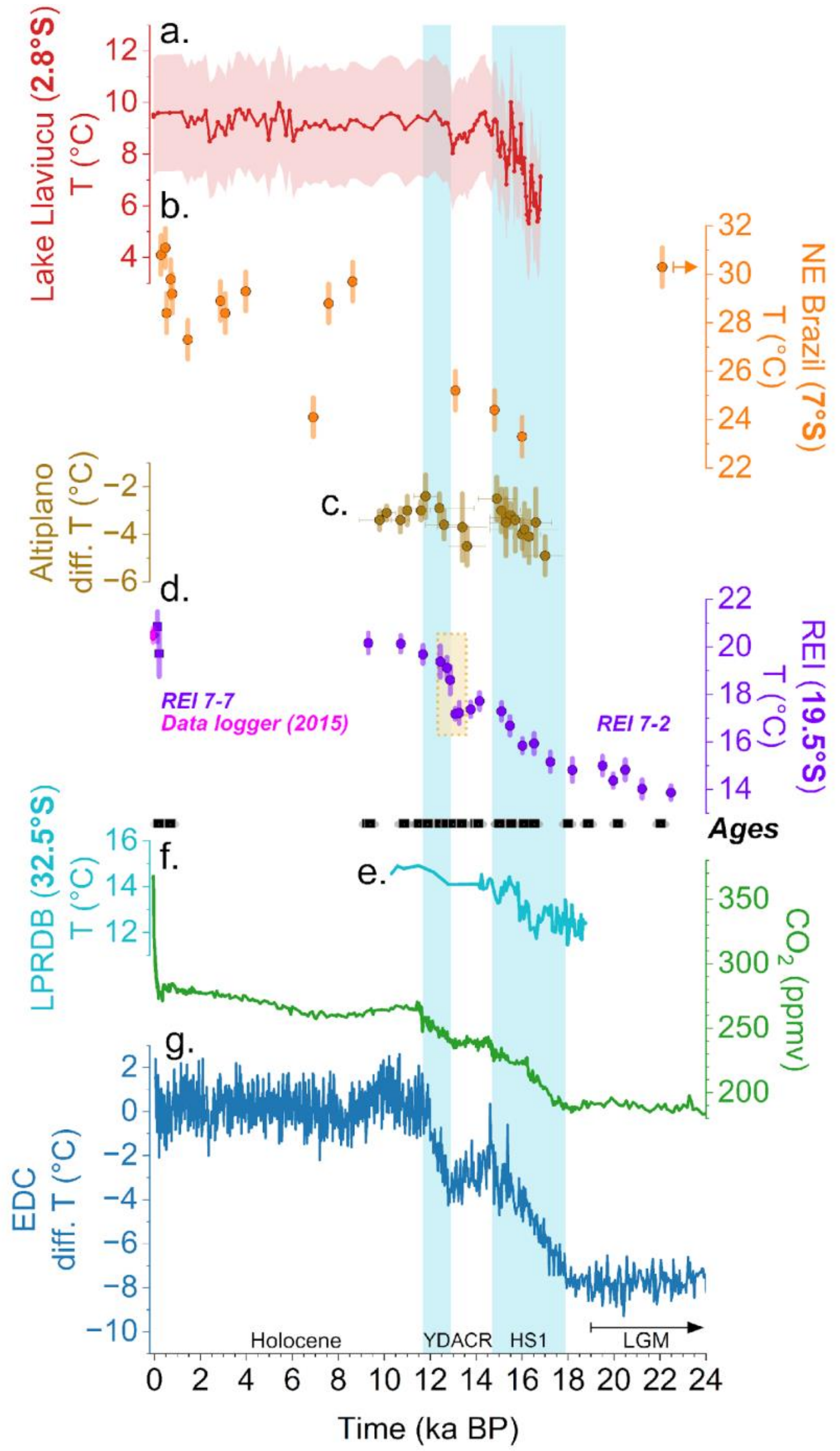

Figure 2. Deglacial temperature reconstructions in South America compared with Antarctic temperature and global atmospheric CO_2. Proxy-based temperature reconstructions in South America from (a) Laguna Llaviucu derived from organic biomarkers (Zhao et al., 2024); (b) Northeastern Brazil derived from noble gases in groundwater, where the orange arrow indicates that the date is likely older (Stute et al., 1995); (c) Temperature relative to present from glacier equilibrium lines from the Andes, within the Altiplano region of Bolivia (Martin et al., 2020); (d) Rei do Mato microthermometry temperature from stalagmite REI 7-2 (purple circles), measured at the top of stalagmite REI 7-7 (purple squares), average cave temperature measured in 2015 (20.5 °C, magenta diamond) and U-Th dates (black squares) (this study). The yellow dashed rectangle indicates the interval of most abrupt warming; (e) La Plata River Drainage Basin (LPRDB) GeoB6211-2 derived by methylation of branched tetraether (MBT')- and cyclization of branched tetraether (CBT)-based mean air temperature (Chiessi et al., 2015). Antarctic records of (f) CO_2 concentration (Bereiter et al., 2015) and (g) temperature anomalies at EPICA Dome C (EDC) site (Landais et al., 2021).

L. 180 There appears to be a strong ACR response, which is also seen in other regional records, yet not much is said about it.

Reply: We thank the reviewer for drawing attention to this important feature of the temperature evolution. In response, we have revised the paragraph (lines 186-191) to highlight the cooling of the ACR and the consistency with other regional records.

Lines 186-191: "*Beyond the overall glacial-interglacial temperature difference, our record reveals pronounced multi-centennial variability characterized by a two-step warming pattern, interrupted by a cooling phase of about 0.5 ºC during the ACR. This cooling is also consistent with Andean glacier history (Jomelli et al., 2014) and lake records from the Andes of Ecuador (Zhao et al., 2024).*"

Fig. 3 Help the reader by adding arrows indicating wetter or drier.

Arrows added.

Original:

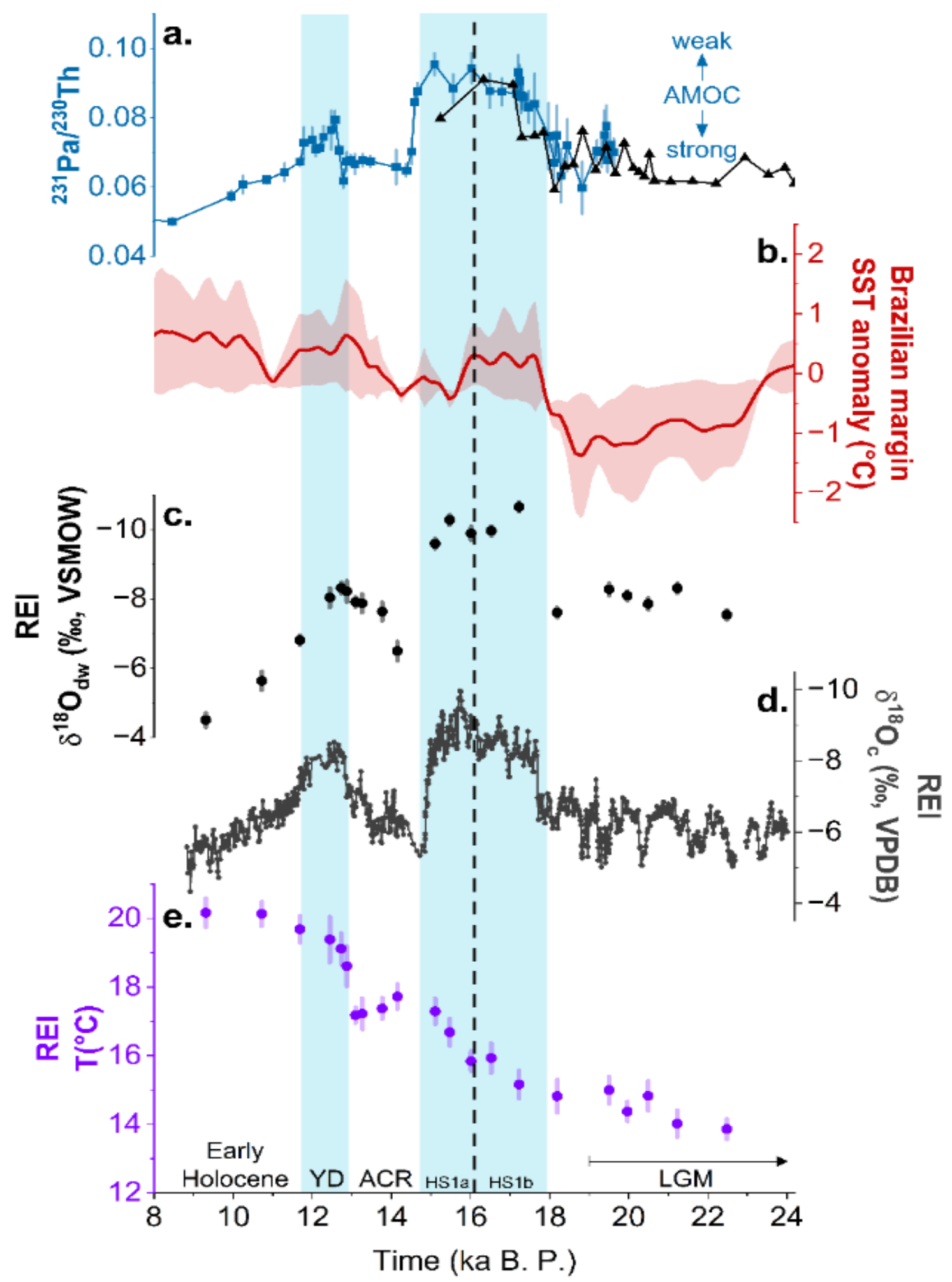

Modified:

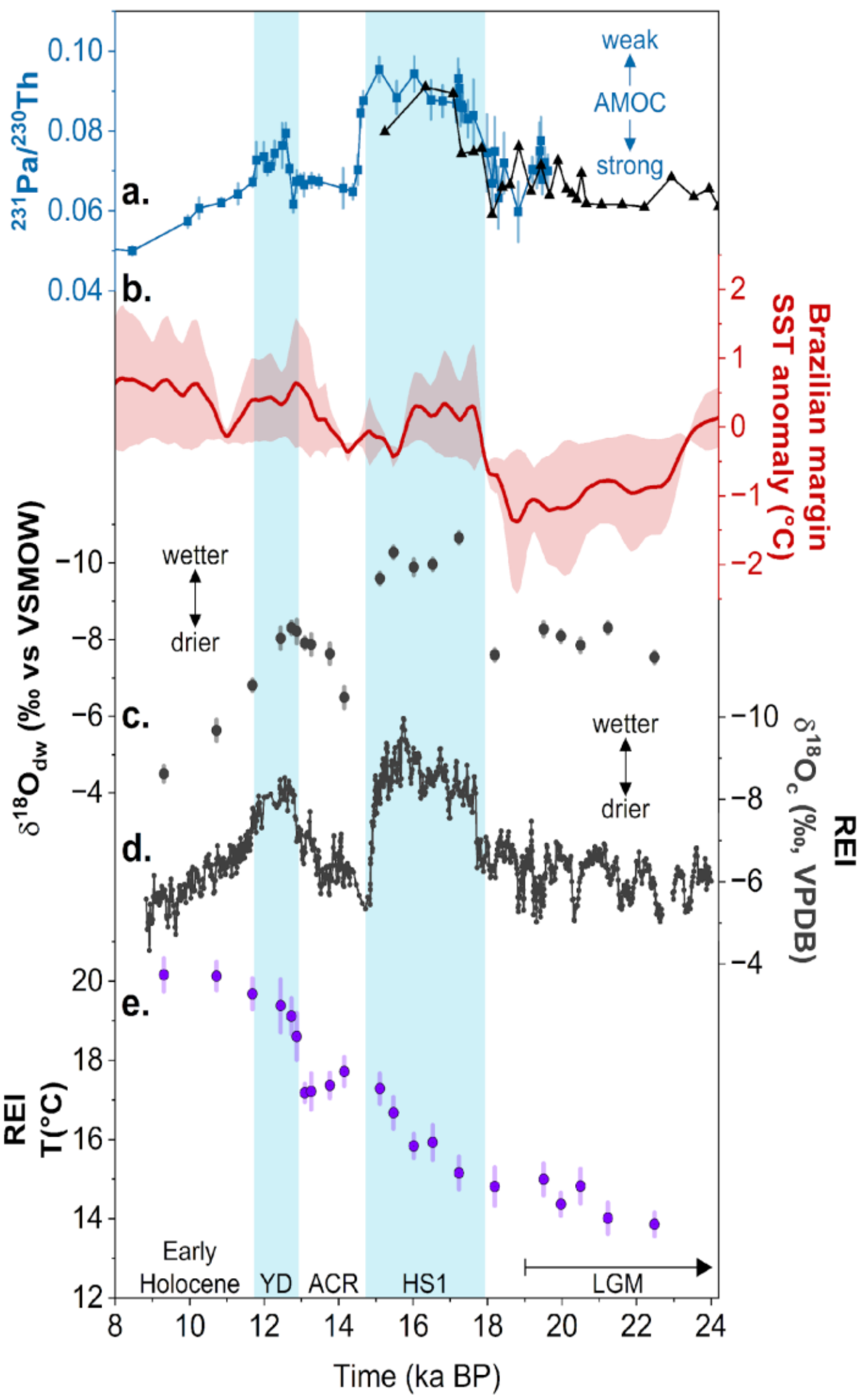

L. 346 delete 'In order"

Deleted. "*In order to measure liquid-vapor homogenization temperatures*…" was replaced with "*To measure liquid-vapor homogenization temperatures*…" (line 364).

Reviewer #2 (Remarks to the Author):

This is an interesting study of temperature changes in the South American tropics coincident with periods of rapid climatic change during the last deglaciation. The method used to reconstruct paleotemperatures (nucleation-assisted fluid inclusion microthermometry) is rapidly gaining traction and appears to provide robust and precise values. The dating of the stalagmite appears solid, as evidenced, in part, by only small changes between ages prior to and following the detrital Th correction, the small errors, and the stratigraphic consistency. The other paleotemperature reconstructions from the region allow important points of comparison, as does the CMIP6 downscaled climate model simulation.

As I read it, the most important goal of this research is to allow a point of comparison (periods of rapid warming during the last deglaciation) for projected future climate changes. To me, this gets a little lost in the weeds, first because the writing could be tightened, and second because climate model boundary conditions are not sufficiently defined. The authors do a good job of explaining that temperature change during the YD and HS1 was not driven solely by GHG but also by AMOC, cloud cover, etc. But if we are comparing past warming to projected future warming, why not also quantify changes in GHG? They are rising much faster today than they did during the last deglaciation. This should be quantified. Likewise, why focus solely on projections of climate? **Why not using well documented warming of the last several decades (presented in Fig 1d) as a point of comparison?** The results presented in this study ring an alarm bell. This area of South America is warming faster today (and will continue to do so even under optimistic emissions scenarios) than it did during periods of massive reorganizations in the circulations of the oceans and atmosphere.

9 – since you are linking elevated CO2/temperatures during deglaciation to elevated CO2/temperatures today and in the future, perhaps quantify the magnitude and rate of CO2 increase from the LGM to early Holocene and then provide note that it is exceeded by the magnitude and rate of anthropogenic emissions over the last century. You must be careful not to ascribe all of the warming to CO2 because, as the authors note, complex feedbacks, many of which are difficult to capture in climate model simulations, also play important roles. But CO2 certainly plays a role so quantifying it, while also providing these caveats, seems like an important component of the discussion.

Reply: The reviewer raises an excellent point about the role of GHG forcing. We intentionally focused on the rates of temperature change and the associated mechanisms (AMOC, cloud cover) because the primary aim of this paper was to present the new temperature reconstruction and document the natural variability and rates of change during the last deglaciation. However, we agree with the reviewer that the **climate response to CO_2 forcing** is a critical context for our findings. To address this, we made edits to lines 206-216, highlighting that warming rates during the ACR-YD are much slower than current warming, making the observed modern warming trends particularly alarming.

Lines 206-216: "*The Rei do Mato record might be used as a benchmark for future projections of regional warming over central-eastern South America. In this context, it is interesting to compare the period of strongest deglacial warming in our record (upper range of 0.123 °C/decade) with the transient temperature response of 0.32 °C/decade (Fig. 1d) since 1980, driven by an increase in the global atmospheric CO_2 concentration of 18.9 ppm/decade between 1980 and 2025 (Lan et al., 2026). This comparison shows how warming rates in recent decades far exceed the fastest rates we documented during the last deglaciation in this region. Given that the temperature response to a CO_2 increase is transient and feedbacks have not yet reached an equilibrium with CO_2 forcing, further increases in the rate of warming are expected in the coming decades until the climate system approaches equilibrium.*"

The reviewer is correct that the instrumental record (Fig. 1d) powerfully demonstrates that the region is already warming at an extraordinary pace. To strengthen this idea we added a sentence (lines 192-193) highlighting that the rates of warming observed in recent decades in this region, which are already comparable to, or even exceeding the fastest rates we document during the last deglaciation:

Lines 192-193: "The last warming phase features the fastest warming rate recorded in the Rei do Mato speleothem."

Reference:

Lan, X., Tans, P. and K.W. Thoning: Trends in globally-averaged CO2 determined from NOAA Global Monitoring Laboratory measurements. Version Thursday, 05-Mar-2026 10:03:59 MST https://doi.org/10.15138/9N0H-ZH07

13 – is it a lack or a paucity? Perhaps also precise temperature records? Also, I'm not sure what "in-situ" refers to here.

Reply: "*Paucity*" is more accurate in this case. By 'in situ,' we refer to comparisons between continental and marine records based on terrigenous material. While stalagmites and lake sediments provide local temperature estimates, marine records integrate signals from a broad area, where the influence of different regional compartments remains poorly understood. Nevertheless, we agree that this point is not essential, and we have revised the sentence accordingly (line 13).

Original: *"…and the lack of in-situ paleo-temperature records."*

Replaced with: "*…and the paucity of precise paleo-temperature records.*"

14 – delete comma

Deleted

15 – delete "21st century"

Deleted.

24 – I know it's redundant, but perhaps write "projections of future long-term"

Edited (lines 22-23).

Original: "*still slower than projections of long-term global average warming rates*"

Replaced with: "*still slower than projections of future long-term warming…*"

33 – delete "the" before Heinrich?; 34 – replace dashes on this line with commas

Edited (lines 31-35)

Original: "…*it is punctuated by millennial excursions concomitant with cold events in the Northern Hemisphere – the Heinrich Stadial 1 (HS1) and the Younger Dryas (YD) – and in Antarctica – the Antarctic Cold Reversal (ACR) – linked with periods of weakening and strengthening in the Atlantic Meridional Overturning Circulation (AMOC), respectively.*"

Replaced with: "…*it is punctuated by millennial excursions concomitant with cold events in the Northern Hemisphere, such as Heinrich Stadial 1 (HS1) and Younger Dryas (YD); and in Antarctica, the Antarctic Cold Reversal (ACR). These events are linked with periods of weakening and strengthening in the Atlantic Meridional Overturning Circulation (AMOC), respectively.*"

43 – delete "in South America"

Deleted (line 41).

Original: *"…with profound consequences for tropical ecosystems in South America.*"

Replaced with: "…*with profound consequences for tropical ecosystems.*"

51 – "ice sheet/albedo"?

In that line, I did mean ice-sheet albedo (lines 48-53):

"*Indeed, global climate reanalysis supports the notion that radiative forcing combining greenhouse gases (primarily CO_2) and **ice-sheet albedo** feedbacks explains more than 90 % of the mean global surface temperature evolution during the deglaciation, while 3.5 % can be attributed to AMOC-driven bipolar seesaw dynamics and orbitally-controlled high-latitude insolation changes (Osman et al., 2021)."*

62 – delete "at most on multi-centennial timescales"

Deleted (line 61).

65 – "bear", not "bare"; 65 – perhaps just "often bear uncertainties exceeding 2˚C"

Edited (line 62-64).

Original: *"…where precise temperature reconstructions are rare and often bare significant uncertainties with errors exceeding 2 °C."*

Replaced with: *"…where precise temperature reconstructions are rare and often **bear** uncertainties exceeding 2 °C."*

89 – have you compared historical temperature data from the cave site (or a neighboring town/weather station) to those across the broader region? Do they move in tandem? It would help to prove that there are no microclimatic effects impacting the site.

Reply: We thank the reviewer for this suggestion. Temperatures move in tandem across most of the continent. The spatial correlation between mean annual temperatures at the grid point of Rei do Mato and temperatures across South America, using t2m temperatures from ERA5 for 1980-2024, indicates positive correlations ($r>0.3$, $p<0.05$) across most of the tropics and subtropics, with the strongest correlations at areas surrounding Rei do Mato.

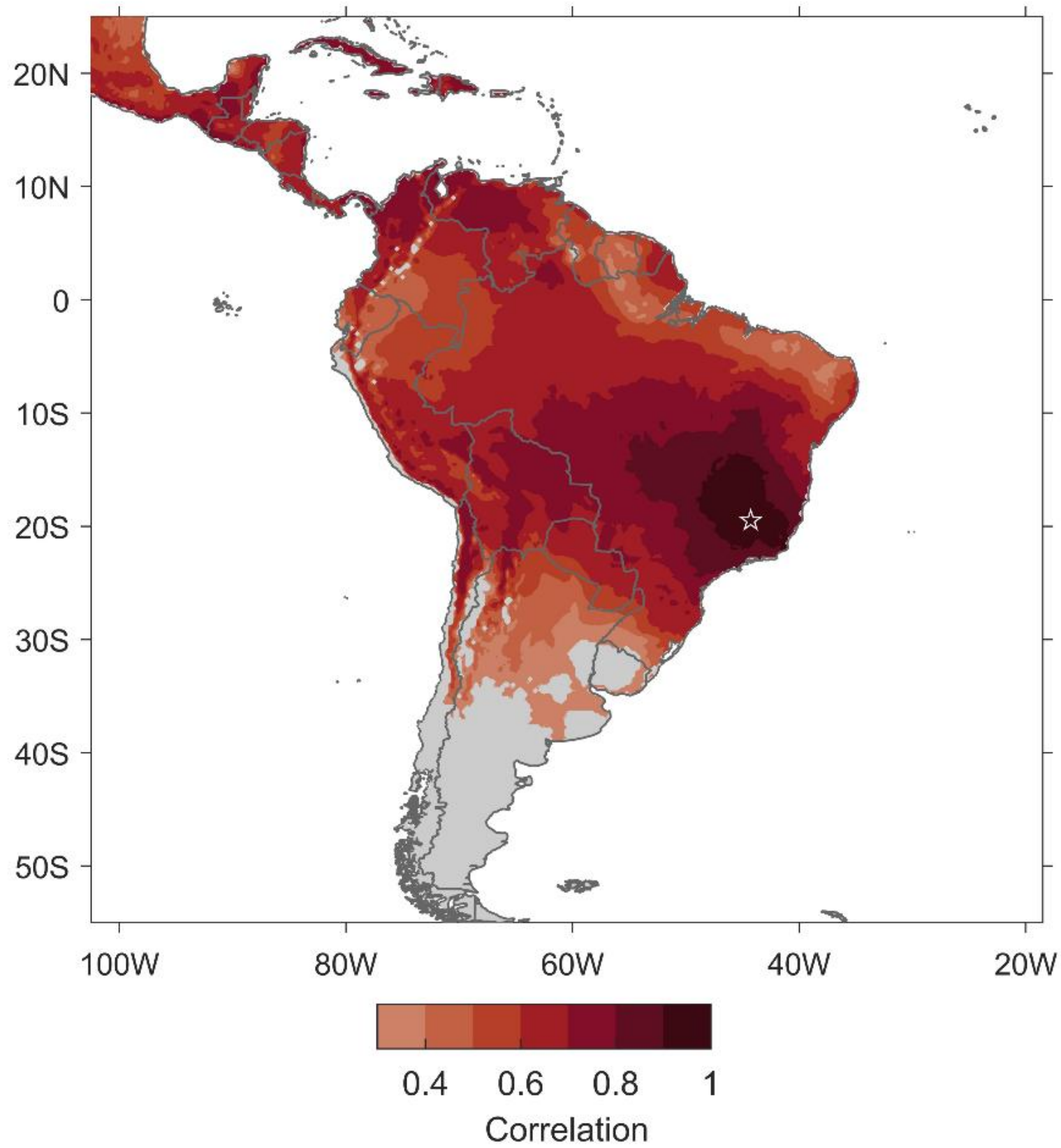

92 – "By applying…"

"*Applying*…" replaced with "*By applying*…" (line 92).

102 – what is the size of the opening? Which direction does the opening face? Are there bats in the cave today?

Reply: The cave opening is about 10 m wide, but the actual access to the walkway is 1-1.5 m. The entrance faces southeast, as depicted in the figure below. There are bat colonies, but not in the area open to tourism.

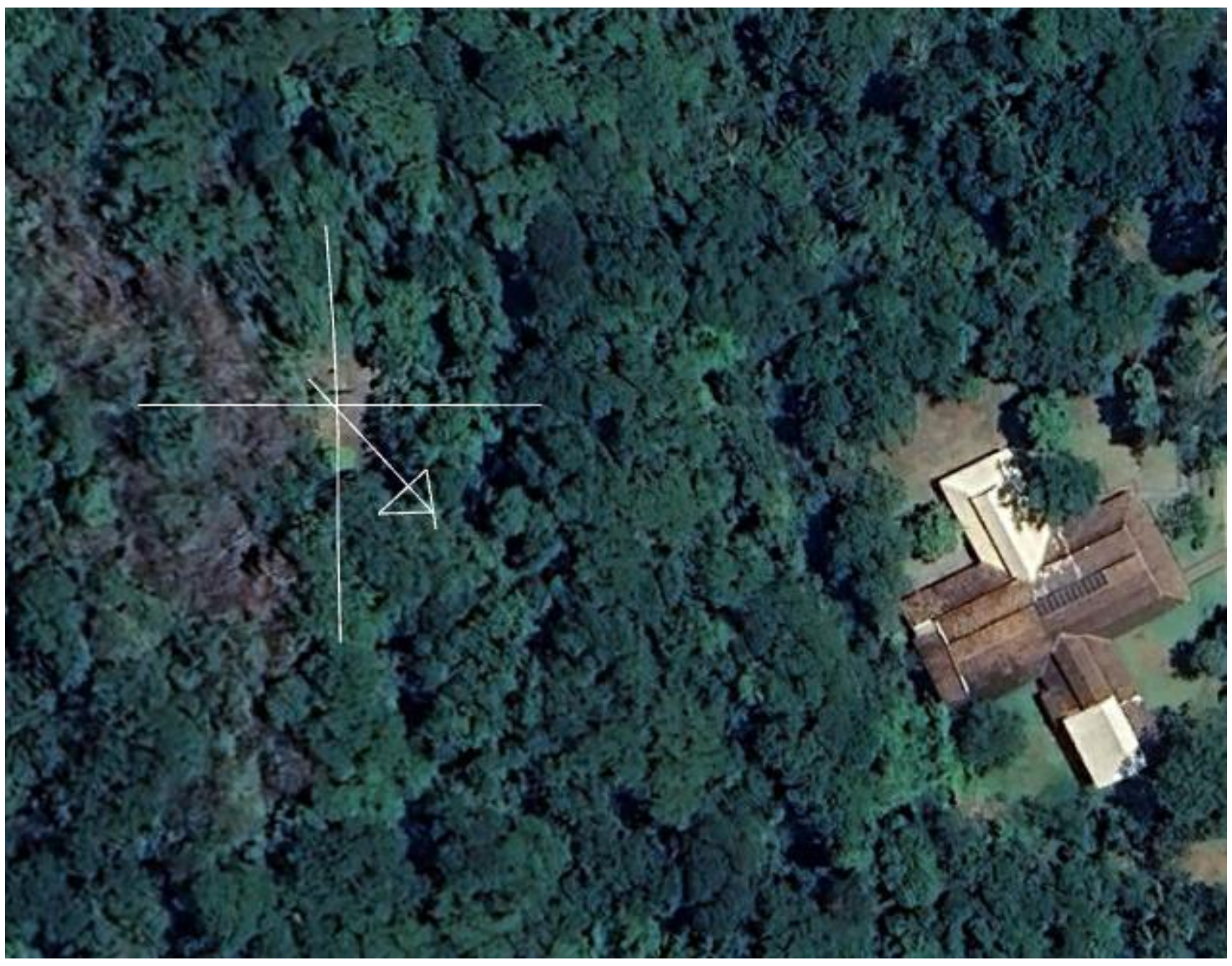

114 – "21.3 ˚C"

"*21.31 °C*" replaced with "*21.3 °C*" (line 119)

115 – delete "for 19 months"

Deleted

117 – is the range in temperature?

Yes, it is. "*reaching 0.6 °C*" was replaced with "*within a range of 0.6 °C.*" (line 122).

117 – delete "high"

Deleted.

119 – do drips (and stalagmite deposition) continue throughout the dry season? This is important to know because if not, then your reconstruction is biased toward warmer, dry season cave temperatures.

Reply: We thank the reviewer for this observation. Most precipitation occurs during the warm season from October to March, when rainfall exceeds 100 mm per month, and is limited during the cold-dry season. Dripping is variable during the dry season. It is significantly reduced and may stop completely if the previous rainy season is relatively dry, whereas when the rainy season is wet, dripping continues through the following dry season. However, cave temperatures are stable year-round, fluctuating within a 0.6 °C

range; therefore, even if there were a bias, it is unlikely to be significant.

121– in a shallow cave, could evaporative cooling near the entrance play a role in determining cave temperature? Under periods of changing monsoon rainfall, this could potentially influence cave temperature.

Reply: We thank the reviewer for bringing this to our attention. Although evaporative cooling likely occurs, its effect on cave temperatures is not significant. Our monitoring indicates that temperatures inside the cave, where the stalagmite was collected, are stable compared with those at the entrance. Moreover, seasonal fluctuations in the cave (0.6°C) are within the uncertainty of the temperature measurements (±0.4°C).

Where in the cave were the stalagmites collected?

Reply: Originally, this information was in the supplementary text 1. Recognizing that this information is very relevant, it was moved to the main manuscript, to the section titled "***The context of the study site***". The information is included in lines 109-116:

"The cave is located on a hillside, with a single entrance on the upper level, and consists of a large hall that extends about 220 meters downward toward the innermost section, which is approximately 30 m below the entrance level (Supplementary Fig. 1). The overlaying bedrock does not exceed 30 m. In 2009, the stalagmite sample REI 7-2 was found broken in a pile of speleothems located near the end of the tourist walkway that traverses the entire hall (Supplementary Fig. 1). Although the exact location where the stalagmite formed is unknown, we assume it originally formed near where it was collected."

146, "(2011), which"

Included "," (line 151)

155 – I'm unclear what a growth band is in this context

Reply: It refers to stalagmite layers. For clarity, the text was edited (lines 160-170).

Original*: "We determined stalagmite formation temperatures from 21 growth bands covering the period from 22.5 to 9.3 ka B.P. The thickness of the individual growth bands ranges between 1 and 5 mm…"*

Replaced with (lines 160-170): *"We determined stalagmite formation temperatures from 21 subsamples across different segments (layers) of stalagmite REI 7-2, selected according to the chronology profile. This provides a mean sampling resolution of approximately 600 years, covering the period from 22.5 to 9.3 ka BP (thousand years before present). Additionally, two temperatures measured at the top of a modern stalagmite (REI 7-7) collected near our deglacial record (REI7-2), match observed temperatures during 2015, supporting the accuracy of the microthermometry temperatures at Rei do Mato cave (Fig. 2d and Supplementary Fig. 5).*

The thickness of the individual layers ranges from 1 to 5 mm, corresponding to intervals of 40-200 years based on the average stalagmite growth rate of ~26µm/year (Supplementary Fig. 3).”

169 – It would help to be able to start by reporting the modern cave temperature as determined through this microthermometry method and comparing it to the measured temp. Are there actively growing (or quite young) samples that have been analyzed?

Reply: We analyzed a younger sample and will now include it. Stalagmite REI 7-7 spans part of the Holocene and modern times, but here we present only two temperatures from its top. These new datapoints were added to Figure 2, along with the measured temperature in 2015. Additional information on REI 7-7 is provided in supplementary figure S5.

Lines 163-167: “*Additionally, two temperatures measured at the top of a modern stalagmite (REI 7-7) collected near our deglacial record (REI7-2), match observed temperatures during 2015, supporting the accuracy of the microthermometry temperatures at Rei do Mato cave (Fig. 2d and Supplementary Fig. 5).*”

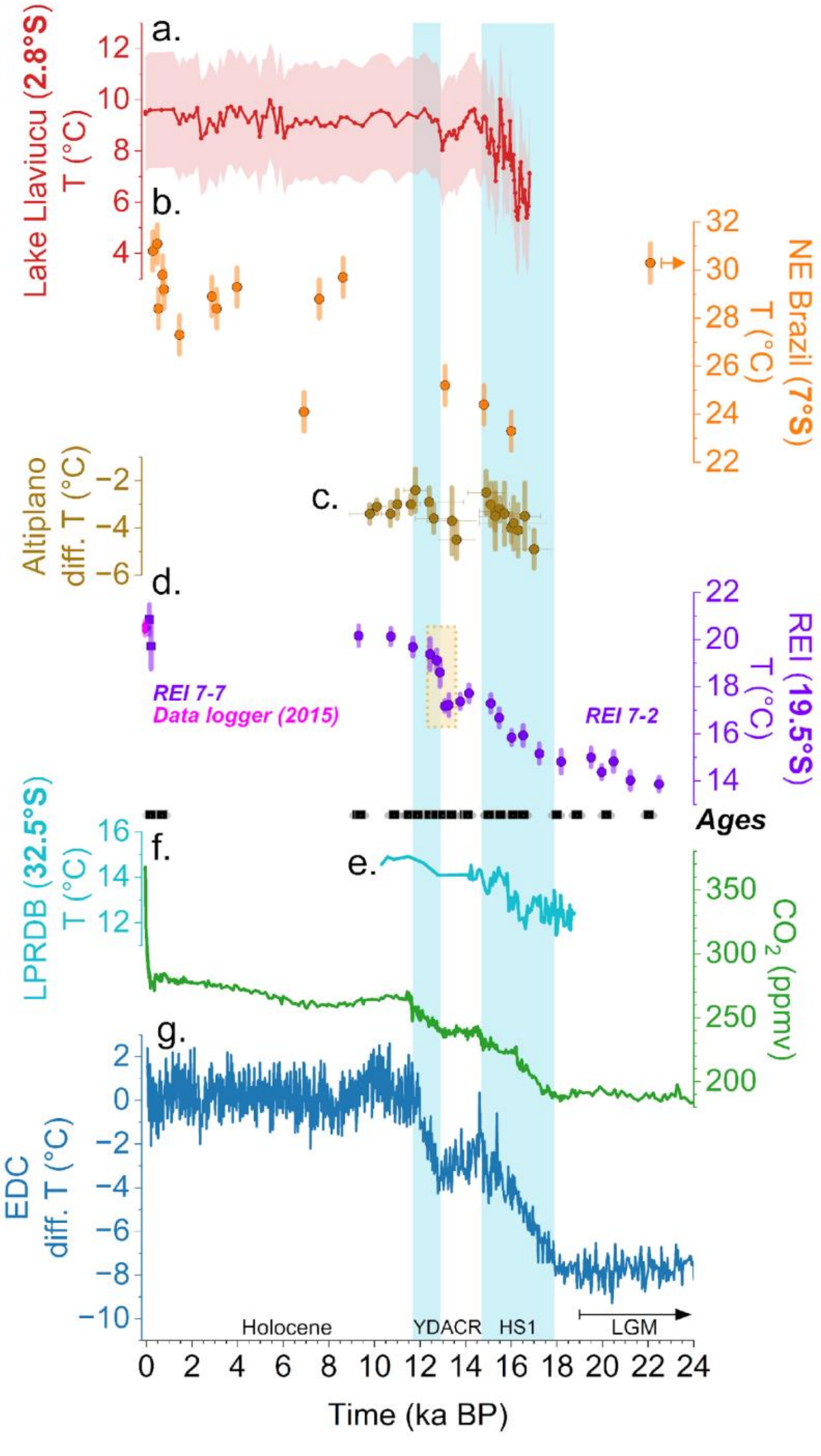

Figure 2. Deglacial temperature reconstructions in South America compared with Antarctic temperature and global atmospheric CO_2. *Proxy-based temperature reconstructions in South America from (a) Laguna Llaviucu derived from organic biomarkers (Zhao et al., 2024); (b) Northeastern Brazil derived from noble gases in groundwater, where the orange arrow indicates that the date is likely older (Stute et al., 1995); (c) Temperature relative to present from glacier equilibrium lines from the Andes, within the Altiplano region of Bolivia (Martin et al., 2020); (d) Rei do Mato microthermometry temperature from stalagmite REI 7-2 (purple circles), measured at the top of stalagmite REI 7-7 (purple squares), average cave temperature measured in 2015 (20.5 °C, magenta diamond) and U-Th dates (black squares) (this study). The yellow dashed rectangle indicates the interval of most abrupt warming; (e) La Plata River Drainage Basin (LPRDB) GeoB6211-2 derived by methylation of branched tetraether (MBT')- and cyclization of branched tetraether (CBT)-based mean air temperature*

(Chiessi et al., 2015). Antarctic records of (f) CO_2 concentration (Bereiter et al., 2015) and (g) temperature anomalies at EPICA Dome C (EDC) site (Landais et al., 2021).

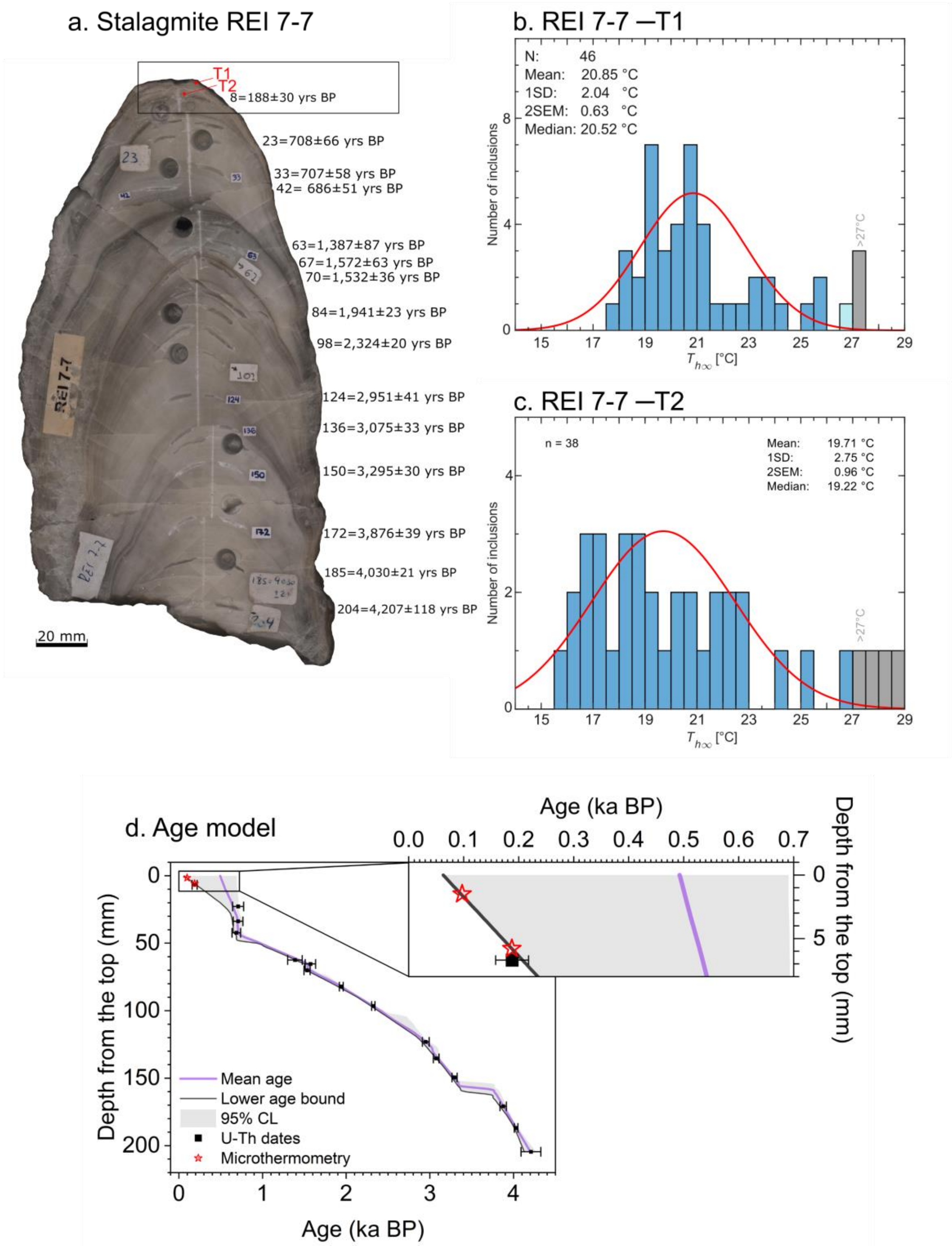

***"Supplementary Figure 5.** (a) Stalagmite REI 7-7 with (b and c) microthermometry measurements and (d) age model. The location of the temperature measurements (T1 and T2) is shown with red stars in (a) and (d). T2 is located near the first measured U-Th age, which overlaps with the lower age boundary (black line) calculated with StalAge. Thus, the age for T1 was also defined by the lower age boundary."*

172 – noble gas-derived

Added "-" (line 183).

178 – I'm not sure of how the specific conventions of Nature Communications, but do you need to define "ka B.P." here?

Reply: To avoid ambiguity, we have defined it in the text as follows: "*ka BP (thousand years before present)*" (line 163).

191 – it would be helpful to provide a few specific examples of glaciers that retreated rapidly at this time. Perhaps plot them on the map in Figure 1?; 192 – perhaps "where AMOC-driven precipitation increases offset melting, leading to glacial advances"

Reply: The glacial retreat was widespread across the tropical Andes. Even in the Altiplano, which experienced precipitation increases during the YD, as evidenced by the positive water balance of Coipasa Lake, for instance. Glacial retreat was the general rule, except for some localized glacier advances. In the manuscript, this part was rewritten for clarity, as follows:

Lines 200-205: "*The YD warming marks a major shift in glacier dynamics in the tropical Andes, as glaciers exhibited a pronounced and widespread retreat during this interval, despite AMOC-driven increases in precipitation in the Peruvian and Bolivian Andes (Palacios et al., 2020; Jomelli et al., 2014). Following the YD, the retreat of most tropical glaciers continued into the early Holocene, when they reached their pre-industrial extent (Palacios et al., 2020).*"

196 – 23rd century?; 197 – can you contextualize the SSP2-4.5 scenario by providing the magnitude of increase in atmospheric GHG: e.g., 1 ppm CO2 equivalent per year?; 198 – ditto for SSP5-8.5 scenario; 200 – "projections from phase 6"

Reply: We thank the reviewer for this suggestion. We originally compared our results with scenarios projections that extend until the year 2300, but after this review, we decided to only consider current warming trends. Significant modifications were thus made in this section:

Lines 206-216: "The Rei do Mato record might be used as a benchmark for future projections of regional warming over central-eastern South America. In this context, it is interesting to compare the period of strongest deglacial warming in our record (upper range of 0.123 °C/decade) with the transient temperature response of 0.32 °C/decade (Fig. 1d) since 1980, driven by an increase in the global atmospheric CO2 concentration of 18.9 ppm/decade between 1980 and 2025 (Lan et al., 2026). This comparison shows how warming rates in recent decades far exceed the fastest rates we documented during the last deglaciation in this region. Given that the temperature response to a CO2 increase is transient and feedbacks have not yet reached an equilibrium with CO2 forcing, further increases in the rate of warming are expected in the coming decades until the climate system approaches equilibrium."

Reference:

Lan, X., Tans, P. and K.W. Thoning: Trends in globally-averaged CO2 determined from NOAA Global Monitoring Laboratory measurements. Version Thursday, 05-Mar-2026 10:03:59 MST https://doi.org/10.15138/9N0H-ZH07

233 – I'm unclear what is meant by "positive bias" here; 240 – the punchline of this paragraph is a bit lost on me

Reply: We thank the reviewer for this comment. The text was edited for clarity (lines 241-251)

Original: "*During the glacial period, cave temperatures were more than 5 °C cooler than today and together with glacial enrichment of seawater $\delta^{18}O$, this results in **a significant positive bias in the $\delta^{18}O_c$ record**. This is revealed by calculated $\delta^{18}O_{dw}$ values, based on $\delta^{18}O_c$ and our cave temperature record, and corrected for the 1 ‰ Glacial – Holocene change in mean ocean $\delta^{18}O$ (Schrag et al., 2002; see Methods), which can serve as a more direct indication of hydroclimate changes compared to classical $\delta^{18}O_c$ records. Our $\delta^{18}O_{dw}$ record does retain most of the millennial scale variability of the $\delta^{18}O_c$ signal, especially around HS1 and during the Holocene. At the onset of the YD, however, the abrupt increase in $\delta^{18}O_c$ that coincides with rapid warming is not reflected in the $\delta^{18}O_{dw}$ record (Fig. 3).*"

Replaced with (lines 241-251): "*Calculated $\delta^{18}O_{dw}$ values based on $\delta^{18}O_c$, our cave temperature record, and corrected for the estimated 1 ‰ Glacial-Holocene change in mean seawater $\delta^{18}O$ (Schrag et al., 2002; see Methods) reveal significant differences from the original $\delta^{18}O_c$ record. During the glacial period, cave temperatures more than 5 °C cooler than today, combined with glacial enrichment of seawater $\delta^{18}O$, produced a significant increase in the $\delta^{18}O_c$ record, independently of rainfall changes. However, our $\delta^{18}O_{dw}$ record retains most of the millennial-scale variability of the $\delta^{18}Oc$, especially around HS1. At the onset of the YD, however, the abrupt increase in $\delta^{18}O_c$ that coincides with rapid warming is not reflected in the $\delta^{18}O_{dw}$ record (Fig. 3). However, in general, when temperatures are known, calculated $\delta^{18}O_{dw}$ can serve as a more direct indication of hydroclimate changes compared to classical $\delta^{18}O_c$.*"

237 – lower sea levels mean substantially more exposed continental shelf on the eastern coast of South America. This could have impacted precipitation (and thus stalagmite) d18O values further inland.

Reply: The exposed continental shelf may have had an influence on the isotope continental effect, ultimately caused by the Rayleigh distillation. However, so far, the only correction that can be estimated based on sea-level variation accounts for the ocean reservoir $\delta^{18}O$, which yields an enrichment of approximately 1‰ (Schrag et al., 2002). Such corrections are addressed in lines 241-251.

277 – "2022), which"

Added "," (line 151).

280 – "the reconstructed temperature"

Original: "*This good correspondence allows us to interrogate the model simulations more closely to determine the driving forces behind the temperature evolution.*"

Replaced with (lines 289-291): "*This good correspondence allows us to investigate the model simulations more closely to determine the drivers behind the reconstructed temperature evolution*."

288 – here you state that the transient model simulations do a good job of capturing the temperature variability reconstructed by the stalagmites. However, in the abstract (line 22), you appear to make the opposite argument.

Reply: We agree with the reviewer that the transient experiment clearly reproduces the millennial- and centennial-scale temperature record in the Rei do Mato stalagmite. We have removed the original statement present in lines 22-23 of the original abstract.

291 – proxy-based

Replaced "*proxy based*" with "*proxy-based*" (line 310).

300 – "the temperature evolution"

Reply: We kept the original version *"…accurate picture of temperature evolution…*" (line 319-320).

399 – replace wrong with inaccurate

Replaced "*…were avoided, as these would lead to wrong ages."* with "*…were avoided, as these would lead to **inaccurate** ages."* (Line 416-417).

400 – "The age model was constructed from the U-series ages using the StalAge software, which allows identification of outliers, …"

Reply: These lines were modified as follows (lines: 418-420):

Original: "*Based on the U-series ages the age model was constructed with the StalAge software (Scholz & Hoffmann, 2011). The method allows to identify outliers, age inversions, hiatuses, and large changes in growth rate.*"

Replaced with: "*The age model was constructed from the U-series ages using the StalAge software, which allows identification of outliers, age inversions, hiatuses, and large changes in growth rate (Scholz & Hoffmann, 2011).*"

Figure 1 – since panel b and d contain purple data points representing temperature, perhaps use a different color for the bars denoting rainfall.

Reply: We thank the reviewer for this suggestion. The color of the bars was changed from purple to green.

Original:

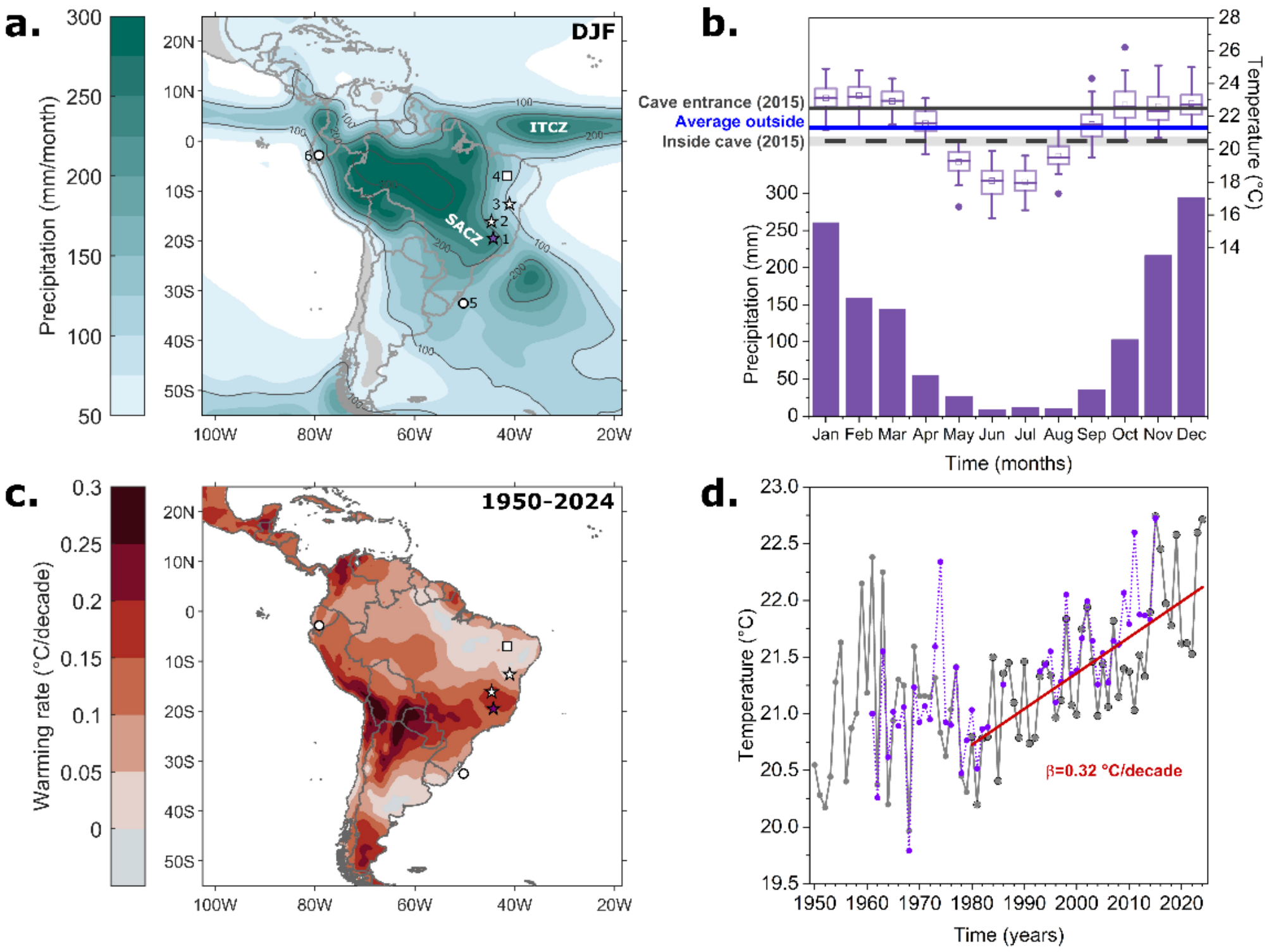

Replaced with:

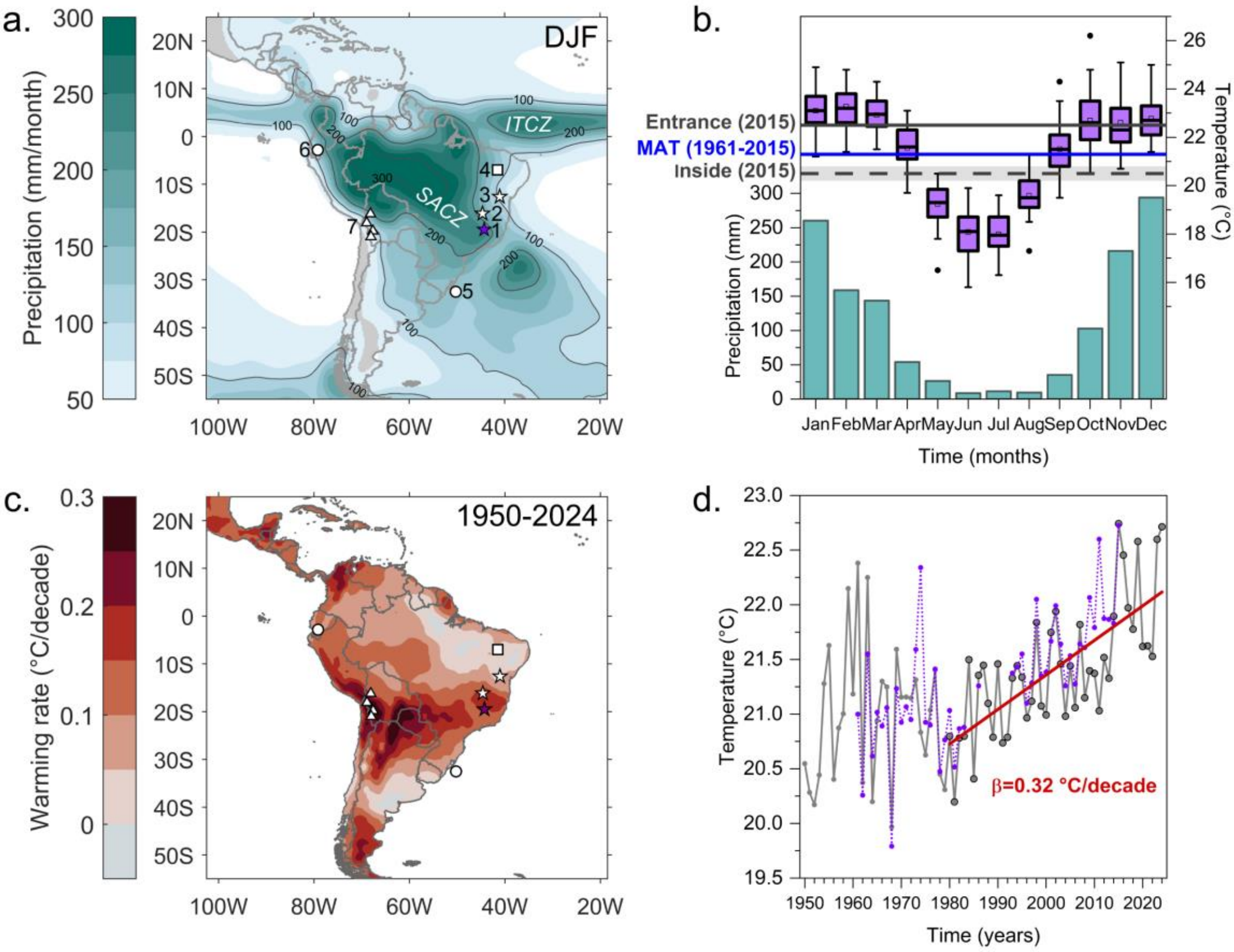

Supplemental Material

7 – tourist cave

Reply: Addressing the comment from reviewer 1, this section was moved to the main manuscript. There, "*touristic*" was replaced with "*tourist*" in line 99.

9 – what are the dimensions of the cave (height, width) and of the opening? Include a cave map. (coming back to this after reading further down the SM: I suggest moving this map up to the intro of the SM).

Reply: We thank the reviewer for the suggestion. The map of the cave was moved up.

33 – delete second comma

Second comma deleted (line 23-24).

Original: "*In contrast, the innermost cave zone, exhibits more stable…*"

Replaced with: "*In contrast, the innermost cave zone exhibits more stable…*"

38 – "19 months of"

Reply: "*The 19-month of…" was replaced* "with "*19 months of…*" (line 28)

43 – why the lag?

Reply: The comment refers to the lines 28-33: "*19 months of continuous cave monitoring at P3 revealed that seasonal temperature variations in the cave atmosphere **lag** the external environment by nearly three to four months. For example, the summer surface temperature peak in October 2015 is observed in the cave only in January 2016, just as the minimum of the surface temperature recorded in May 2015 that is shifted to August in the cave.*"

In this context, the observed lag is likely related to the ventilation dynamics and the thermal inertia of the cave system. The monitoring data indicate more efficient ventilation during the cold season, when surface temperatures fall below the mean cave temperature, promoting cold-air inflow. Likely, as cold air is trapped inside the cave, thermal equilibration with the surface is slow due to limited exchange with the outside. As a result, cave temperatures respond slowly to surface warming and increase with a lag relative to surface temperatures.

68 – delete comma

Reply: Comma deleted (line 70).

Original: "*The stalagmite was cut into two halves along the growth axis**,** with a diamond wire…"*

Replaced with: "*The stalagmite was cut into two halves along the growth axis with a diamond wire…"*

105 – delete comma

Reply: Comma deleted (line 149)

Original: "*Løland et al., (2022)…"*

Replaced with: "*Løland et al. (2022)…"*

168 – "from Slab 2. (b)…"

Reply: Dot added (line 105).

Original: "*projected from Slab 2 (b) Slab 2 of the stalagmite…*"

Replaced with: "*projected from Slab 2. (b) Slab 2 of the stalagmite…*"

The data table showing U/Th isotope ratios contains some font mistakes in the headings.

Reply: Heddings corrected

Sample Number	^{238}U (ppb)		^{232}Th (ppt)		^{230}Th / ^{232}Th (atomic x10^{-6})		d^{234}U* (measured)		^{230}Th / ^{238}U (activity)		^{230}Th Age (yr) (uncorrected)		^{230}Th Age (yr) (corrected)		d$^{234}U_{Initial}$** (corrected)		^{230}Th Age (yr BP)*** (corrected)	
REI7-2-1	60.2	±0.1	1190	±24	593	±12	7507.2	±5.27	0.7106	±0.0044	9397	±60	9331	±76	7707	±6	**9264**	**±76**
REI7-2-11	57.1	±0.1	198	±4	3537	±72	7856	±8.78	0.745	±0.0016	9463	±23	9452	±24	8069	±9	**9394**	**±24**

Reviewer #3 (Remarks to the Author):

The manuscript presents a new speleothem record from a cave in south-eastern Brazil. Speleothems are now widely recognized as valuable paleoclimate archives, as they have the potential to provide unbiased and accurately dated records of changes in annual mean surface air temperature and the hydrological cycle. As South America is not yet well covered by such records, this new record is a valuable addition to the study of regional climate change during the last glacial termination. However, regardless of how good an individual paleoclimate record is, it is just one record and is likely to show local climate variability. Therefore, it is surprising that the authors based their very bold statements, such as claiming to have found a "significant nonlinear response of the climate system to radiative and freshwater flux forcings", on a single record. It is unclear what the authors mean by "response of the climate system" (annual mean temperature?) and how they concluded that the response to radiative forcing (from greenhouse gases?) is strongly nonlinear. The issue of the climate response to freshwater forcing is discussed below.

Reply: We thank the reviewer for this important and incisive comment, which raises important questions about the interpretation of our single-site record and the terminology we have used.

The reviewer correctly notes that any individual paleoclimate record contains local signals, and we agree that caution is warranted when extrapolating to a broader-scale. However, we wish to clarify that our claim regarding "non-linear responses of the climate system" is specifically anchored in independent complementary lines of evidence that collectively support a regional-scale interpretation:

First, our temperature record is broadly consistent with other independently derived temperature reconstructions from tropical South America, including noble gas records from northeastern Brazil (Stute et al., 1995), biomarker records from the Ecuadorian Andes (Zhao et al., 2024), and glacier-based reconstructions from Peru and Bolivia (Martin et al., 2020; Palacios et al., 2020) (Fig. 2). This spatial coherence suggests that our record captures a regional temperature signal rather than purely local cave-specific variability.

Second, the millennial-scale structure of our record, particularly the two-step warming pattern during HS1 and the abrupt warming at the YD onset, aligns with well-documented features of the deglacial climate system, including Antarctic temperature variability (Landais et al., 2021), atmospheric CO_2 changes (Bereiter et al., 2015), and AMOC reconstructions (McManus et al., 2004; Böhm et al., 2015) (Fig. 3).

In summary, we understand that global representation of the temperature evolution cannot be reconstructed from a single record and that some of the original writing was misleading. Therefore, we have revised the text to ensure clarity and accuracy in the following lines:

Original: "*However, our results also point to significant non-linear responses of the climate system to radiative and freshwater flux forcings during the deglacial period expressed by intervals of strongly accelerated warming, underestimated in recent transient paleoclimate modeling experiments.*"

Replaced with (Lines 18-24): "*Here, we show an abrupt regional warming across the Antarctic Cold Reversal-Younger Dryas (ACR-YD) transition, linked to the weakening of*

the Atlantic Meridional overturning circulation (AMOC) that leads to heat build-up in the Southern Hemisphere. Notably, the most rapid deglacial temperature increase recorded at our cave was still slower than projections of future long-term warming, highlighting the unprecedented nature of the current greenhouse gas forcing."

Lines 326-331: *"Nonetheless, the Rei do Mato record also reveals an interval of non-linear regional temperature response to GHG forcing, observed during the ACR-YD transition. Given the robust nature of our cave record, we provide a crucial benchmark for understanding temperature evolution in central-eastern South America. This study highlights the need for additional precise paleotemperature records from other regions where different climate factors may be at play."*

My second problem with the manuscript under consideration is that, unlike the authors, I cannot see two rapid warming events in their data. There is no doubt that the reconstructed temperature rise at 13 ka is abrupt, if not nearly instantaneous. However, the reported abrupt warming between 16 ka and 15 ka is attributed solely to a single data point at around 16 ka. If this outlier is excluded, the best statistical model for the period between 18 ka and 14 ka is a linear temperature rise, which is fully consistent with a similar rise in CO_2 concentration (see also their Figure S7). A small temperature decline during the ACR period can also be easily explained by the stabilization of CO_2 during this time, as well as the "bipolar seesaw". Thus, the only mysterious aspect of the new record is the abrupt warming at the beginning of the Younger Dryas, with temperatures rising by around 2°C in just one hundred years. Such abrupt and strong warming cannot be explained by greenhouse gases or the classical bipolar seesaw, given the timescale of the Southern Hemisphere response. Therefore, it must be a very localized effect. The cited paper by Pedro et al. (2018) shows the appearance of a very local warming spot just 100 years after the AMOC shut down, not far from the location of the cave. However, this result is unlikely to be robust, as it is not evident in other simulations of AMOC shutdown. In any case, regardless of whether this abrupt warming is real, it cannot be considered a large-scale phenomenon. Furthermore, it is unclear what the authors meant by "tipping point" (line 190) in the context of their study.

Reply: We thank the reviewer for their observations and agree with them; we have modified the manuscript accordingly, highlighting only the abrupt event at the ACR-YD transition.

Moreover, we recognize that the temperature record presented here does not reflect a large-scale response, but rather likely captures the regional temperature evolution in central eastern South America. The abrupt temperature increase is reproduced by the TraCE 21k experiment, as presented in Figure 4.

Also, "tipping point" was misused. That line was edited as follows:

Lines 200-205:

"*The YD warming marks a major shift in glacier dynamics in the tropical Andes, as glaciers exhibited a pronounced and widespread retreat during this interval, despite AMOC-driven increases in precipitation in the Peruvian and Bolivian Andes (Palacios et*

al., 2020; Jomelli et al., 2014). Following the YD, the retreat of most tropical glaciers continued into the early Holocene, when they reached their pre-industrial extent (Palacios et al., 2020).”

Concerning the climate response to the freshwater flux (FWF): When comparing the results of the TraCE21Kii experiments performed with the CCSM3 model (mistakenly referred to as the “TraCE21Kii model” by the authors), it should be noted that the freshwater forcing used in these experiments (as well as in the initial TraCE21K experiment by Liu et al. 2009) is completely inconsistent (in fact, inverted) with the global ice sheet reconstructions, the fact recognized by the paleoclimate modelling community already for a long time. Real reconstructions of the freshwater flux into the North Atlantic (see, for example, Kapsch et al., 2022, Figure 1), derived from ice sheet evolution reconstructions (Tarasov et al. 2012; Peltier et al. 2015), show a maximum in FWF during the warm Bolling interval and after the Younger Dryas, with a rather small FWF during the Heinrich 1 and Younger Dryas periods. This is the opposite of the FWF scenario used in the TraCE21Kii experiment. Thus, when the authors compare their results with those of TraCE21Kii, they compare real climate change with the FWF scenario designed by Liu et al. (2009) and He & Clark (2022) solely to achieve the best fit between their climate model results and paleoclimate reconstructions. As the actual FWF during the last glacial termination differs significantly from that used in TraCE21Kii, the discussion of the role of freshwater flux in climate change during past glacial terminations (pages 14 and 15) is fundamentally flawed.

References:

Kapsch, M.-L., Mikolajewicz, U., Ziemen, F., & Schannwell, C. (2022). Ocean response in transient simulations of the last deglaciation dominated by underlying ice-sheet reconstruction and method of meltwater distribution. Geophysical Research Letters, 49, e2021GL096767.

Liu, Z., Otto-Bliesner, B.L., He, F., Brady, E.C., Tomas, R., Clark, P.U., Carlson, A.E., Lynch-Stieglitz, J., Curry, W., Brook, E. and Erickson, D., (2009). Transient simulation of last deglaciation with a new mechanism for Bølling-Allerød warming. Science, 325, 310-314.

Peltier, W. R., Argus, D. F., & Drummond, R. (2015). Space geodesy constrains ice age terminal deglaciation: The global ICE-6G_C (VM5a) model. Journal of Geophysical Research: Solid Earth, 120(1), 450–487.

Tarasov, L., Dyke, A. S., Neal, R. M., & Peltier, W. (2012). A data-calibrated distribution of deglacial chronologies for the North American ice complex from glaciological modeling. Earth and Planetary Science Letters, 315–316, 30–40.

Reply: We thank the reviewer for this critical and constructive evaluation of the freshwater flux (FWF) forcing used in the TraCE21Kii experiments. We appreciate the correction regarding the model nomenclature (CCSM3 vs. "TraCE21Kii model") and the pointer toward the physical inconsistencies between the TraCEKii FWF scenario and ice-sheet reconstruction.

We acknowledge that the FWF applied in TraCE21Kii is not a physical reconstruction of meltwater discharge, but rather a numerical mechanism designed to "hose" the model

into an AMOC state that matches paleoclimate proxies. Following the reviewer's feedback, we consulted with the authors of the TraCE21Kii study (He & Clark, 2022). They clarified that the FWF in their experiment serves as a proxy to prescribe the ***meridional ocean heat transport*** associated with AMOC changes, rather than representing an accurate ice-sheet meltwater budget.

The reviewer is correct that discussing this as a "response to freshwater flux" is misleading if the flux itself is inverted relative to reality. Consequently, we have revised the manuscript to shift the focus from **freshwater forcing** to **AMOC-driven climate response**. By reframing our comparison in terms of AMOC-induced changes in heat transport, we believe it remains highly valuable for understanding the climate's sensitivity to ocean circulation shifts, regardless of the specific trigger (be it meltwater or other internal dynamics).

Edits were made:

Original:

*"**Non-linear temperature response to CO2 forcing and AMOC***

The cave temperature reconstruction is overall consistent in magnitude and timing with the transient model simulation of surface temperature from TraCE21Kii (He and Clark, 2022) which combines greenhouse gases (GHG), freshwater flux (FWF), ice sheet (ICE) and orbital (ORB) forcings (Fig. 4a, b). This good correspondence allows us to interrogate the model simulations more closely to determine the driving forces behind the temperature evolution. In the model, central-eastern South America warms more than the rest of the continent through the deglacial period (Fig. 4a)."

Replaced with (lines 285-300):

*"**Temperature response to CO2 and AMOC forcings***

The cave temperature reconstruction is overall consistent in magnitude and timing with the transient model simulation of surface temperature from the TraCE21Kii experiment (He and Clark, 2022), performed with the Community Climate System Model version 3 (CCSM3) (Collins et al., 2006; Yeager et al., 2006). This good correspondence allows us to investigate the model simulations more closely to determine the drivers behind the reconstructed temperature evolution.

The TraCE21Kii experiment combines greenhouse gases (GHG), ice sheet (ICE) and orbital (ORB) forcings, and AMOC-induced changes in ocean heat transport (AMOC) (Fig. 4a, b). Although the latter is often referred to as freshwater forcing (FWF), it should be noted that in the TraCE21Kii simulations, this is not intended as a realistic reconstruction of meltwater flux, which is known to be physically inconsistent with certain ice-sheet reconstructions (e.g., Kapsch et al., 2022). Instead, the FWF in TraCE21Kii serves as a numerical driver to prescribe an AMOC state that matches paleoclimate records (Supplementary Fig. 10). Our comparison, therefore, evaluates the temperature response to these prescribed AMOC-driven changes in heat transport."

References:

Collins, W. D. et al. The Community Climate System Model version 3 (CCSM3). *Journal of Climate* **19**, 2122–2143 (2006). https://doi.org/10.1175/JCLI3761.1

Kapsch, M.-L. et al. Ocean response in transient simulations of the last deglaciation dominated by underlying ice-sheet reconstruction and method of meltwater distribution. *Geophysical Research Letters* **49**, e2021GL096767 (2022).

Yeager, S. G. et al. The low-resolution CCSM3. *Journal of Climate* **19**, 2545–2566 (2006). https://doi.org/10.1175/JCLI3744.1

Response to referees

Reviewer #1 (Remarks to the Author):

I believe that the authors have done a good job of responding to referees criticisms, and this paper while being inconsistent in its findings with other that show more gradual warming will trigger further inquiry.

I suggest the authors tweak the sentence on line 82 where they seem to suggest that it is odd that the Fuquene record should be responding to Greenland. Overall the dominant pacing of long records north of about 11oS, i.e. Lakes Junin and Fuquene and speleothems Santiago cave and Cueva del Diamante, all of which show AMOC forcing and are therefore in synch with Greenland rather than Antartica. Sites south of this line (which probably shifts between 9-11oS according to glacial stage) are more likely to show Antarctic forcing (Bradbury 2001)

Full and late glacial lake records along the PEP1 transect: Their role in developing interhemispheric paleoclimate interactions

J. P. Bradbury, M. Grosjean, S. Stine and F. Sylvestre

In: Interhemispheric Climate Linkages, edited by V. Markgraf

Academic Press 2001

Reply: We thank the reviewer for this comment. While in is true that tropical precipitation usually responds to the NH millennial events, the temperature response is different, aligned with Antarctic temperatures. This was also observed in a cave record from Borneo, located at 4°S (Loland et al., 2022). Since Lake Fúquene is the only record in that region of Colombia, and it bares significant uncertainty, the original sentence was meant to present that study, without taking that statement for granted, but also, without completely disregarding it. Yet the sentence was modified to made is more appropriate:

Original: "*The exception are perhaps the Colombian Andes, where temperature reconstructed from Lake Fúquene (5°N) pollen was suggested to follow the pacing from the Greenland / North Atlantic region.*"

Replace with: "*An exception was found in the Colombian Andes, where temperature reconstructed from Lake Fúquene (5°N) pollen was suggested to align with the Greenland / North Atlantic millennial events.*"

Reference:

Løland, M. H., Krüger, Y., Fernandez, A., Buckingham, F., Carolin, S. A., Sodemann, H., Adkins, J. F., Cobb, K. M., and Meckler, A. N.: Evolution of tropical land temperature across the last

glacial termination, Nat. Commun., 13, 5158, https://doi.org/10.1038/s41467-022-32712-3, 2022.

Reviewer #2 (Remarks to the Author):

I reviewed an earlier version of this manuscript and expressed several concerns about the arguments being made. The authors are now to be commended for addressing these concerns in detail. The paper now reads quite well, and I feel it is ready for publication.

Reply: We thank the reviewer for this comment.

Reviewer #3 (Remarks to the Author):

I am fully satisfied with the author's response to my criticism, and I would recommend the manuscript for publication in Nature Communications.

Reply: We thank the reviewer for this comment.
