# Supplementary material for: Rapid warming in South America during the last deglaciation
Source: Nat Commun. 2026 Jun 20;17:8068. doi: 10.1038/s41467-026-74093-x (PMC13454564; doi:10.1038/s41467-026-74093-x)
Supplement: Supplementary file 3 [file 41467_2026_74093_MOESM1_ESM.docx]

**Supplementary information**

**Rapid warming in South America during the Last Deglaciation**

A. Ampuero G.^1,2^*, N. M. Stríkis^1^, A. N. Meckler^3,4^, Y. Krüger^3^, M. Vuille^5^, H. Vonhof^2^, L. Pasqualetto^3^, V. C. Mayta^6^, N. M. M. Medina^1^, G. Utida^7^, H. Zhang^8^, H. Cheng^8^, R. L. Edwards^9^, L. E. P. Travassos^10^, P. Piacsek^1^, T. K. Akabane^1^, F. W. Cruz^1^*

^1^Institute of Geosciences, University of São Paulo, São Paulo, Brazil

^2^Department of Climate Geochemistry, Max-Planck Institute for Chemistry, Mainz, Germany

^3^Department of Earth Science, University of Bergen, Bergen, 5007, Norway

^4^Bjerknes Centre for Climate Research, Bergen, 5007, Norway

^5^Department of Atmospheric and Environmental Sciences, University at Albany, State University of New York, Albany, NY, USA

^6^Department of Climate and Space Science and Engineering, University of Michigan, Ann Arbor, MI, USA

^7^Institute of Astronomy, Geophysics and Atmospheric Sciences, University of São Paulo, São Paulo, Brazil

^8^Institute of Global Environmental Change, Xi’an Jiaotong University, Xi’an, 712000, China

^9^Department of Earth and Environmental Sciences, University of Minnesota, Minneapolis, MN, USA

^10^Department of Geography, Pontifical Catholic University of Minas Gerais, Minas Gerais Brazil

*Corresponding authors: [angela.ampuero@mpic.de](mailto:angela.ampuero@mpic.de), cbill@usp.br

**Supplementary text 1: Cave monitoring**

We used Testo 175H1 data loggers to record air temperature (accuracy of ± 0.4 °C) and relative humidity (accuracy of ± 2 %). One data logger was placed at the cave entrance (P1), and three were installed in the cave hall: one in the middle zone (P2) and two in the innermost, lowest zone (P3 and P4), close to the site where the stalagmites were recovered (Supplementary Fig. 1). Monitoring data were recorded from January 2015 to July 2016 with some interruptions in different loggers. While this monitoring period is too short to establish a reliable transfer function between surface and cave air temperatures, it provides valuable insights into the seasonality of the cave climate and the relationship between cave and surface conditions.

Relative humidity inside the cave fluctuates between 94 and 100 %, reaching values close to 100 % during the rainy season (Supplementary Fig. 2a). These conditions are stable compared with seasonal fluctuations outside, which range from 26 to 96 %.

Temperatures recorded at the cave entrance (P1) closely track surface air temperatures from the ERA5-Land reanalysis and observations at the Sete Lagoas station (Supplementary Fig. 2b). In contrast, the innermost cave zone exhibits more stable temperature conditions, with a seasonal amplitude of 0.6 °C. This is minimal compared to the 14.3 °C amplitude observed in P1, highlighting the cave's strong buffering capacity. Despite the small amplitude, the seasonal temperature variations recorded in the innermost cave zones offer valuable insights into the processes governing subsurface heat exchange. 19 months of continuous cave monitoring at P3 revealed that seasonal temperature variations in the cave atmosphere lag the external environment by nearly three to four months. For example, the summer surface temperature peak in October 2015 is observed in the cave only in January 2016, just as the minimum of the surface temperature recorded in May 2015 is shifted to August in the cave.

From February to December 2015 (the common period of continuous measurements between P1 and P3), the average outside air temperature was 22.5 °C at P1 and 20.5 °C at P3. This indicates a 2 °C cold bias inside the cave relative to the outside. This temperature offset might be related to a delay in the signal transfer from the surface into the cave (cf. refs^1,2^). However, given the cave geometry – a single entrance opening into a hall that slopes downward – we assume that the ventilation regime of the cave likely promotes a cold-trap effect, with preferential airflow into the cave during the cold season, thereby substantially lowering the cave air temperature below the mean surface air temperature. A dominant role of the ventilation regime on the cave temperature is also supported by pronounced seasonal fluctuations of around 0.6 °C and a clear temperature gradient within the cave from P2 to P3.

**Supplementary Figure 1.** **Plan view map of the cave and elevation profiles**. Red circles indicate the monitoring sites along the touristic walkway: entrance (P1), the middle section (P2), and the innermost area (P3 and P4). Sample REI 7-2 was collected near P3 and P4, at about 28 m below the entrance level. Elevation profile A-A illustrates the height difference between P1 and P2, whereas profile B-B begins near P2 and indicates the position of P3 and P4.

**Supplementary Figure 2. Monitored relative humidity and air temperature from January 2015 to July 2016.** Records within the cave hall, at locations P2, P3, and P4 (a, b), and the same records compared to the observations at the cave entrance at P1 (c, d). (d) Includes a monthly averaged record from Sete Lagoas station (OMM_83586) (magenta line) and a record of daily values from ERA 5-Land reanalysis^3^ (light blue line). Data from ERA5 is available by the CC-BY 4.0 license <https://creativecommons.org/licenses/by/4.0/>.

**Supplementary text 2: Samples and sample preparation**

Our record, spanning LGM to the Early Holocene, was derived from stalagmite REI 7-2, which is 385 mm long and covers the period from ~24 to 8.8 ka BP (Supplementary Fig. 3). The stalagmite has an average growth rate of 25.5 ± 5.6 µm/year and is dominated by a columnar, open calcite fabric, indicative of high, stable drip rates^4,5^. Fluid inclusions are abundant throughout most of the stalagmite and are distributed in layers of higher and lower abundance. The inclusions are monophase liquid and slightly elongated parallel to the crystal *c*-axis. The volumes of the analyzed inclusions range from 10^2^ to 10^5^ µm^3^ (Supplementary Fig. 6).

The stalagmite was cut into two halves along the growth axis with a diamond wire saw (Well 6500). Then, two 10 mm-thick slabs were cut from one of the half pieces parallel to the growth axis. The first slab (closer to the growth axis, Slab 1) was polished and used for dating and the stable isotope profile (Supplementary Fig. 3a). The second slab (Slab 2, Supplementary Fig. 3b), adjacent and parallel to the first slab, was used for the microthermometric measurements. 3 x 2 cm blocks were cut out from the center of Slab 2 and used to prepare, first, a ∼100 µm-thick polished section for petrographic analysis and then a second ∼300 µm-thick unpolished section for microthermometry. While epoxy resin was used to attach the sample to a standard 28x48 mm glass slide for cutting the petrographic section, superglue was used for the microthermometry section. For cutting the sections, a low-speed rock saw (Buehler Isomet) was used to minimize mechanical stress on the calcite. The microthermometry section was finally cut into 5 mm wide stripes with a small diamond wire saw (Well 3500) and subsequently immersed in acetone to dissolve the superglue and remove the section from the glass substrate. Targeted fragments with a maximum diameter of 5 mm were split from the stripes, cleaned with acetone, and coated with a thin film of immersion oil to reduce light scattering from the unpolished surface, making the samples transparent for visual observation of the fluid inclusions under the transmitted-light microscope. The remaining pieces of the stalagmite are preserved in the archive at the University of Sao Paulo.

Since the samples for stable isotopes and microthermometry were collected on different slabs with slight offsets of the age-depth relation, we established separate age models for each slab (Supplementary Fig. 3c-d). We initially sampled Slab 1 for dating, and the layers were visually traced to their corresponding ages in Slab 2, based on the distinct characteristics of the stalagmite banding. We refined the Slab 2 age model by directly sampling for dating within this slab, targeting the identified intervals of most rapid warming.

**Supplementary Figure 3. Image of stalagmite sample REI 7-2 with age models.** (a) Slab 1 of the stalagmite. This is a 1 cm thick slab cut from one of the halves of the stalagmite. The stippled purple line indicates the transects drilled for stable isotope analyses along the main growth axis. Gray lines highlight the location of the samples drilled for U-Th, with the ages and errors indicated on the right. Additional samples that were drilled, but not analyzed. Light blue lines indicate U-Th dates visually projected from Slab 2; (b) Slab 2 of the stalagmite, taken immediately behind Slab 1. Blocks were cut along the growth axis, from which the petrographic and the microthermometry sections were prepared. Photographs of the latter are superimposed on the Slab 2 image. Yellow lines indicate the locations of samples analyzed by laser-assisted microthermometry, with yellow numbers denoting sample identifiers. As in (a), gray and light blue lines indicate drilled samples for U-Th and visually projected dates from Slab 1, respectively; (c) StalAge age model for Slab 1 and d) for Slab 2. Note the detailed chronological analysis carried out between 14 and 12 ka BP in the REI-7-2 speleothem, done to better delineate the abrupt warming observed during the transition between the ACR-YD transition. The model excludes the bottom age from Slab 1 because this section was not preserved in Slab 2 due to the stepped fracture plane that formed when the stalagmite was removed from the original formation site in the cave. ACR Antarctic Cold Reversal, YD Younger Dryas.

**Supplementary Figure 4.** **Distributions of calculated T_h∞_ values derived from microthermometric measurements of 21 growth bands of stalagmite REI 7-2**. Each cave temperature represents the average T_h∞_ of ~40 individual fluid inclusions (dark blue bars). The panels indicate the sample size (N), the mean value, 1SD, and 2 SEM of the T_h∞_ distribution. Outliers were excluded from the statistical analysis using a 4-MAD criterion (light blue bars). Note that inclusions with T_h(obs)_ > 27 °C are not shown in the graphs.

**Supplementary Figure 5. Stalagmite REI 7-7 with age model and microthermometry measurements.** (a) Stalagmite REI 7-7 with (b, c) microthermometry measurements and (d) age model calculated with StalAge. The location of the temperature measurements (T1 and T2) is shown with red stars in (a) and (d). Gray bars indicate inclusions with T_h(obs)_ > 27 °C in (b) and (c).

**Supplementary Figure 6.** **Selection of fluid inclusion images from stalagmite REI 7-2, illustrating the shapes and sizes of the fluid inclusions in different sub-samples.** All panels (a - l) show an overview image of the selected inclusion in the initial monophase liquid state with a 40x objective, and an image of the inclusion in the two-phase state, taken with the 100x objective for subsequent measurements of the vapor bubble radius. Scale bars are shown in panel (a). FI Fluid Inclusion

**Supplementary text 3: Methods of calculation**

***Calculation of drip water δ^18^O_dw_***

To obtain the δ^18^O_c_ of the stalagmite segments, between 2 and 4 chunks of calcite were taken as near as possible from the location where the microthermometry measurements were performed in Slab 2. After homogenization of the calcite powders with a cleaned mortar and pestle, we took two aliquots from each sample and stored them in cleaned 12 ml borosilicate vials with septum caps for isotope measurements. We consider the average value to represent the isotope composition of the stalagmite growth layer. As shown in ref^6^ we calculated δ^18^O_dw_ applying the inverted temperature calibration^7^ based on our average growth layer δ^18^O_c_ and our cave temperature record. We applied the correction for a 1 ‰ change in mean ocean δ^18^O from the LGM to the Holocene^8^, assuming a linear relationship with global sea-level changes^9^. We used a Monte Carlo approach (N=100,000) to propagate errors from δ^18^O_c_, temperatures, and sea level changes. To account for δ^18^O_c_ uncertainties, we used 1SD of the individual measurements at each stalagmite growth band. For sea level uncertainty, we used the 1SD of the sea level data points within the time interval covered by each temperature data point.

***Stepwise regression***

Stepwise regression analyses were performed with the “stepwiselm” function in Matlab R2023a, which iteratively adds or removes predictors based on builds a linear regression model by adding and removing predictors based on *p*-value and F-test criteria. In our analysis, a predictor was added if the *p*-value was below 0.05 and removed if it was above 0.10. As predictors, we used surface temperatures from single forcing experiments (GHG, ICE, AMOC and ORB), and our proxy-based temperature record as the response. To match the temporal resolution, the high-resolution model data were smoothed with a 100-year moving average and resampled to the proxy chronology, taking the mean value within the range of chronological uncertainty. We first produced a regression model for the whole time series, considering the interval where all predictors and response overlap (19-8 ka BP). A second regression model was then calculated, focusing on the ACR-YD abrupt transition.

***Breakpoint detection and linear regression analysis***

We identified significant shifts in the linear trends of the cave temperature record by applying a “trend fitting method” using code from ref^10^, with some modifications to account for the data uncertainty (2SEM) (supplementary code 1). The method requires setting the maximum number of breakpoints in advance. To assess the sensitivity of the identified breakpoints, we performed several tests with varying maximum breakpoint counts. The segments between the breakpoints are modeled independently using a Monte Carlo approach, in which 1000 trends were computed for each segment, accounting for data uncertainty. The final angular coefficient for each segment represents the mean slope of the 1000 regressions within the 95% confidence interval (Supplementary Fig. 7 and Supplementary Table 1). The statistical significance of the trend was assessed using a two-tailed Monte Carlo test. We first performed these analyses using the complete temperature record and then for specific time intervals, where the largest warming rates were detected.

**Supplementary Figure 7.** **Temperature change rates estimated by identifying breakpoints and linear trends.** The angular coefficients (β) indicate the temperature change rate, in °C/ century (a) considering the complete record; and (b) the interval from the ACR onwards, where we identify the largest warming trend of the deglacial period (β = 0.66). All results are shown in Supplementary Table 1. ACR Antarctic Cold Reversal, S Segment

| Period | Segment | Time interval (years BP) | β (°C/century) | β low (°C/century) | β high (°C/century) | p-value |
| --- | --- | --- | --- | --- | --- | --- |
| All | S1 | 9306 - 13090 | 0.06 | 0.08 | 0.03 | <0.01 |
|  | S2 | 13090 - 14150 | -0.05 | 0.03 | -0.12 | 0.24 |
|  | S3 | 14150 - 19504 | 0.06 | 0.07 | 0.04 | <0.01 |
|  | S4 | 19504 - 22481 | 0.03 | 0.06 | 0.01 | 0.02 |
| ACR - Early Holocene | S5 | 9306 - 12872 | 0.04 | 0.07 | 0.01 | 0.01 |
|  | S6 | 12872 - 13090 | 0.64 | 1.23 | 0.05 | 0.03 |

**Supplementary Table 1. Results of the trend fitting analyses.** These results are illustrated in Supplementary Fig. 7. ACR Antarctic Cold Reversal.

**Supplementary Figure 8. Stalagmite records and precipitation anomalies from TraCE21Kii experiment^12^.** (a) Records of ẟ^18^O_c_ from stalagmite from central-eastern South America: Rei do Mato (REI), Lapa Sem Fim (LSF)^11^, and Paixao^11^. (b-d) Precipitation anomalies relative to the pre-industrial period (0-0.5ka) based on Decadal average precipitation from TraCE21Kii experiment^12^. Stars in the maps indicate the location of the caves. Gray vertical bars indicate Northern Hemisphere cooling episodes. YD Younger Dryas, HS1 Heinrich Stadial 1, ACR Antarctic cold reversal, LGM Last Glacial Maximum.

**Supplementary Figure 9. Comparison of precipitation (δ^18^O_dw_) and temperature proxy records.** Records from Borneo^6^ (a, b) and Rei do Mato (c, d; this study), with (a-c) with 2SEM error bars and 1SD error bars (d). Temperature variations are in phase at both sites, whereas precipitation changes are in antiphase, indicating decoupling between the two variables. Gray vertical bars indicate Northern Hemisphere cooling episodes. YD Younger Dryas, HS1 Heinrich Stadial 1, ACR Antarctic cold reversal, LGM Last Glacial Maximum.

**Supplementary Figure 10. Comparison of proxy records and model for AMOC dynamics.** AMOC reconstruction from ^231^Pa/^230^Th from ocean sediment core OCE326-GGC5^13^ (blue squares) and OCE326-GGC5^14^ (green triangles) with internal precision error bars (2SD). Modelled AMOC transport used in TraCE21Kii experiment^12^ (red line). Gray vertical bars indicate Northern Hemisphere cooling episodes. YD Younger Dryas, HS1 Heinrich Stadial 1, ACR Antarctic cold reversal, LGM Last Glacial Maximum, AMOC Antarctic Cold Reversal.

**References**

1.	Badino, G. Underground Meteorology-“What’s the weather underground?” *AC* **39**, 427–448 (2010).

2.	Domínguez-Villar, D., Krklec, K., López-Sáez, J. A. & Sierro, F. J. Thermal impact of Heinrich stadials in cave temperature and speleothem oxygen isotope records. *Quat. res.* **101**, 37–50 (2021).

3.	Muñoz Sabater, J. (2019): ERA5-Land monthly averaged data from 1950 to present. Copernicus Climate Change Service (C3S) Climate Data Store (CDS). DOI: [10.24381/cds.68d2bb30](https://doi.org/10.24381/cds.68d2bb30) (Accessed on 07-JUL-2025)”.

4.	Frisia, S. Microstratigraphic logging of calcite fabrics in speleothems as tool for palaeoclimate studies. *IJS* **44**, 1–16 (2015).

5.	Martín-Chivelet, J., Muñoz-García, M. B., Cruz, J. A., Ortega, A. I. & Turrero, M. J. Speleothem Architectural Analysis: Integrated approach for stalagmite-based paleoclimate research. *Sedimentary Geology* **353**, 28–45 (2017).

6.	Løland, M. H. *et al.* Evolution of tropical land temperature across the last glacial termination. *Nat Commun* **13**, 5158 (2022).

7.	Tremaine, D. M., Froelich, P. N. & Wang, Y. Speleothem calcite farmed in situ: Modern calibration of δ18O and δ13C paleoclimate proxies in a continuously-monitored natural cave system. *Geochimica et Cosmochimica Acta* **75**, 4929–4950 (2011).

8.	Schrag, D. P. *et al.* The oxygen isotopic composition of seawater during the Last Glacial Maximum. *Quaternary Science Reviews* **21**, 331–342 (2002).

9.	Lambeck, K., Rouby, H., Purcell, A., Sun, Y. & Sambridge, M. Sea level and global ice volumes from the Last Glacial Maximum to the Holocene. *Proc. Natl. Acad. Sci. U.S.A.* **111**, 15296–15303 (2014).

10.	Dong, X. *et al.* Coupled atmosphere-ice-ocean dynamics during Heinrich Stadial 2. *Nat Commun* **13**, 5867 (2022).

11.	Stríkis, N. M. *et al.* Timing and structure of Mega‐SACZ events during Heinrich Stadial 1. *Geophysical Research Letters* **42**, 5477 (2015).

12.	He, F. & Clark, P. U. Freshwater forcing of the Atlantic Meridional Overturning Circulation revisited. *Nat. Clim. Chang.* **12**, 449–454 (2022).

13.	McManus, J. F., Francois, R., Gherardi, J.-M., Keigwin, L. D. & Brown-Leger, S. Collapse and rapid resumption of Atlantic meridional circulation linked to deglacial climate changes. *Nature* **428**, 834–837 (2004).

14.	Böhm, E. *et al.* Strong and deep Atlantic meridional overturning circulation during the last glacial cycle. *Nature* **517**, 73–76 (2015).
