# Supplementary material for: Rapid warming in South America during the last deglaciation
Source: Nat Commun. 2026 Jun 20;17:8068. doi: 10.1038/s41467-026-74093-x (PMC13454564; doi:10.1038/s41467-026-74093-x)
Supplement: Supplementary file 4 [file 41467_2026_74093_MOESM3_ESM.zip › Supplementary Code 1.pdf]

```
1 % This collection of MATLAB code is a supplement to the paper:
2 %
3 % Ampuero et al., Rapid Warming in South America during the Last Deglaciation (under revision)
4 %
5 % The code performs the "breakpoint detection" and "trend-fitting" analysis as described in that work.
6 % First, the "breakpoints" are identified and then the "trend-fitting" is
7 % performed in each segment following a Monte Carlo approach.
8 %
9 % To run the code, the user should put the name of .csv file in Line 27.
10 % The file should include three columns (first: time [years]; Second: temperature [°C]; Third: uncertainty [2SEM]) with no title.
11 % The .csv file should be put in the same folder of this code.
12 % The user can set the maximum number of breakpoints (cp_max) in Line 23 and the number of iterations (n_iter) in Line 25.
13 %
14 % Kind regards,
15 %
16 % Angela Ampuero
17
18 %============================================================
19 %% Input
20 %============================================================
21 clear all; clc; clf;
22
23 cp_max=3;   %  The user can set Maximum number of breakpoints
24
25 n_iter = 1000; % number of Monte Carlo iterations
26
27 data = load ('proxy.csv'); % the user should put the name of .csv file here
28
29 t   =   data(:,1); %time [years]
30 x   =   data(:,2); %temperature [°C]
31 err =   data(:,3); % uncertainty (2SEM)
32
33 %============================================================
34 %% Breakpoint detection
35 %============================================================
36
37 id=findchangepts(x,'MaxNumChanges',cp_max,'Statistic','linear');
38
```

```
id=[id;id];
id=sort(id);

id=[1, id', length(x)];% get all the transition positions and start and end points

%============================================================
%% Map configuration
%============================================================

figure('color','white','paperorientation','portrait');
set(gcf, 'Position',  [100, 100, 550, 310])
subplot('Position', [0.1, 0.2, 0.8, 0.7]); % [left, bottom, width, height]

%============================================================
%% Plot original data
%============================================================

errorbar(t, x, err, 'LineStyle', 'none', 'Color', [0.5 0.5 0.5], 'CapSize', 3); %error bar
hold on
plot(t,x,'.','markersize',20); hold on

j=1;
segment_info = table;

for i=1:2:length(id)

  %============================================================
  %% Linear regression (Monte Carlo uncertainty propagation)
  %============================================================

  t_seg = t(id(i):id(i+1));  % segment to model
  t_seg_centered = t_seg - mean(t_seg); % center the timevalues around zero
  x_seg = x(id(i):id(i+1));  % datapoints in the segment

  slopes = zeros(n_iter,1);  %Preallocate arrays
  intercepts = zeros(n_iter,1);  %Preallocate arrays

  for m = 1:n_iter  %Perturb data
```

```
    x_sample = x_seg + randn(size(x_seg)) .* err(id(i):id(i+1)); %add noise
    lm_mc = fitlm(t_seg_centered, x_sample);  %fit to linear model
    slopes(m) = lm_mc.Coefficients.Estimate(2);  %store slopes
    intercepts(m) = lm_mc.Coefficients.Estimate(1);  %store intercepts
end
%=============================================================
%% Plot CI bounds
%=============================================================

% Compute fitted values for each Monte Carlo simulation
mean_t = mean(t_seg);
x_pred_mc = zeros(n_iter, length(t_seg));
for m = 1:n_iter
    unc_intercept = intercepts(m) - slopes(m) * mean_t;  %transforms centered regresssion back to the original time axis
    x_pred_mc(m, :) = slopes(m) * t_seg + unc_intercept; %predicted line for one montercarlo realization
end

% Get the 2.5th and 97.5th percentile across simulations at each time point (95% CI)
x_fit_low = prctile(x_pred_mc, 2.5, 1);
x_fit_high = prctile(x_pred_mc, 97.5, 1);

% Plot shaded uncertainty envelope
fill([t_seg; flipud(t_seg)], [x_fit_low'; flipud(x_fit_high')], [0.7 0.85 1.0], 'EdgeColor','none', 'FaceAlpha', 0.5);

% Compute mean, 2.5th and 97.5th percentiles (95% CI)
slope_mean = mean(slopes);  %average slope across montecarlo simulations
slope_CI = prctile(slopes, [2.5, 97.5]);  % 95% confidence interval
intercept_mean = mean(intercepts);
intercept_CI = prctile(intercepts, [2.5, 97.5]);

%=============================================================
%% Significance test: Monte Carlo p-value (two-tailed Montecarlo test)
%=============================================================

p_value = min(1, 2 * min(mean(slopes <= 0), mean(slopes >= 0)));

%=============================================================
%% Convert centered results back to original time axis
```

```
%============================================================

intercept_mean_uncentered = intercept_mean - slope_mean * mean_t;
intercept_CI_uncentered = intercept_CI - slope_CI * mean_t;

%============================================================
%% Compute final regression line
%============================================================

% Final regression line
x_fit = slope_mean * t_seg + intercept_mean_uncentered;
plot(t_seg, x_fit, 'Color', [0, 0.2, 0.6], 'LineWidth', 2);  % dark blue

% Breakpoint line
line([t(id(i)) t(id(i))], [12 22], 'LineWidth', 0.75, 'Color', [0.3, 0.3, 0.3], 'LineStyle', '--');  % gray dashed

%============================================================
%% Save table
%============================================================

segment_info.Segment(j) = j;
segment_info.Start_Year(j) = t(id(2*j - 1));
segment_info.End_Year(j) = t(id(2*j));
segment_info.Slope_C_per_100yr(j) = slope_mean*100;
% segment_info.Intercept(j) = intercept_mean;
% segment_info.Intercept(j) = intercept_mean_uncentered
segment_info.Slope_CI_Low_per_100yr(j) = slope_CI(1)*100;
segment_info.Slope_CI_High_per_100yr(j) = slope_CI(2)*100;
% segment_info.Intercept_CI_Low_centered(j) = intercept_CI(1);
% segment_info.Intercept_CI_High_centered(j) = intercept_CI(2);
% segment_info.Intercept_CI_Low_uncentered(j) = intercept_CI_uncentered(1);
% segment_info.Intercept_CI_High_uncentered(j) = intercept_CI_uncentered(2);
segment_info.p_value(j) = p_value;

%============================================================

j=j+1;

```

```
end

%============================================================
%% Final plot details
%============================================================


errorbar(t, x, err, 'LineStyle', 'none', 'Color', [0.5 0.5 0.5], 'CapSize', 3); %error bar
hold on
plot(t,x,'.','markersize',20,'MarkerFaceColor',[0.5 0 0.5]); hold on

xlim([min(t)-250 max(t)+250]);
ylim([min(x - err)-1 max(x + err)+1]);
xticks = get(gca, 'XTick');                 % Get current ticks
xticklabels = xticks / 1000;                % Convert to ka
set(gca, 'XTickLabel', xticklabels);        % Set new labels
xlabel('Time (ka B. P.)', 'FontSize', 12);
ylabel('Temperature (°C)', 'FontSize', 12);
title('Breakpoint Analysis of Temperature Record', 'FontSize', 14);
set(gca, 'FontSize', 11);

%============================================================
%% Export image
%============================================================
% name=['linear_trend_all'];
% print('-djpeg', name, '-r850');
```
